# A machine learning approach to economic complexity based on matrix completion

**DOI:** 10.1038/s41598-022-13206-0

**Published:** 2022-06-10

**Authors:** Giorgio Gnecco, Federico Nutarelli, Massimo Riccaboni

**Affiliations:** AXES Research Unit, IMT School for Advanced Studies, 55100 Lucca, Italy

**Keywords:** Applied mathematics, Applied physics, Complex networks

## Abstract

This work applies Matrix Completion (MC) – a class of machine-learning methods commonly used in recommendation systems – to analyze economic complexity. In this paper MC is applied to reconstruct the Revealed Comparative Advantage (RCA) matrix, whose elements express the relative advantage of countries in given classes of products, as evidenced by yearly trade flows. A high-accuracy binary classifier is derived from the MC application to discriminate between elements of the RCA matrix that are, respectively, higher/lower than one. We introduce a novel Matrix cOmpletion iNdex of Economic complexitY (MONEY) based on MC and related to the degree of predictability of the RCA entries of different countries (the lower the predictability, the higher the complexity). Differently from previously-developed economic complexity indices, MONEY takes into account several singular vectors of the matrix reconstructed by MC. In contrast, other indices are based only on one/two eigenvectors of a suitable symmetric matrix derived from the RCA matrix. Finally, MC is compared with state-of-the-art economic complexity indices, showing that the MC-based classifier achieves better performance than previous methods based on the application of machine learning to economic complexity.

## Introduction

Since the early 2000s, building metrics for measuring economic complexity has been a set goal. Starting from the Economic Complexity Index (ECI) developed by Hidalgo and Hausmann^1^, it has become clear how most traditional economic growth theories often shrank internal socio-economic dynamics of countries through strict assumptions, restricting the analysis to a small subset of pre-determined factors. Unlike traditional growth theories, economic complexity measures are based on a data-driven approach and are generally agnostic about the determinants of countries’ competitiveness. For instance, the ECI seeks to explain the knowledge accumulated by a country and expressed in all the economic activities present in that country. More and more refined measures of economic complexity have become available in the last few years. In a recent review, Hidalgo^2^ identifies two main streams of the literature on economic complexity: the first involves metrics of so-called relatedness, whereas the second concerns economic complexity metrics, which apply dimensionality reduction techniques based, e.g., on Singular Value Decomposition (SVD). Metrics of relatedness measure the affinity between an activity and a location, while methods related to dimensionality reduction search for the best combination of factors explaining the structure of a given specialization matrix.

According to the principle of relatedness, the probability that a location *c* (e.g., a country) enters or exits an economic activity *p* (e.g., a sector) is influenced by the presence of related activities in that location. This poses, however, more profound questions about the role played by similar countries in determining the likelihood that the location *c* enters the economic activity *p*. Furthermore, while the principle of relatedness attempts to model the probability of entering an activity *p* by the location *c*, it does not provide hints about whether *c* will enter *p* successfully or not. Besides, there is a strong connection between the concept of production function – a function connecting economic inputs to outputs – and economic complexity via the SVD of a suitable specialization matrix. Indeed, as discussed in Hidalgo^2^, the Cobb-Douglas production function of a given set of outputs might be expressed in terms of the SVD of the specialization matrix. In particular, in that context, the singular vectors represent the factors of production employed to produce a given set of outputs. Having more of such factors contributes to better explain the outputs. A similar idea applies to the case of economic complexity, for which SVD is used to learn the singular vectors (factors) that best explain the structure of a specialization matrix $\mathbf{R} { \in {\mathbb {R}}^{C \times P}}$, where *C* is the number of countries considered in the analysis, and *P* is the number of products examined (at a given aggregation level). The ECI index is closely related to the leading singular vectors of that specialization matrix (Hidalgo^2^), i.e., to a truncated SVD of the matrix. These are also the leading eigenvectors of the product of the specialization matrix with its transpose. Usually, scholars select one of the first two eigenvectors (i.e., the ones associated with the two largest eigenvalues) because it carries out the maximum amount of information. However, this approach has some important drawbacks, as discussed in Morrison et al.^3^. Recently, Sciarra et al.^4^ combined information coming from the first two eigenvectors into a unique index called GENeralised Economic comPlexitY (GENEPY). Nevertheless, it is worth noticing that, by doing this, the other eigenvectors are neglected and, together with them, further information which could potentially better explain economic complexity. Therefore, it looks reasonable to explore a suitable way to carefully select some other most informative eigenvectors (or, more in general, singular vectors) beyond the first two.

In this respect, the present paper exploits a class of machine-learning methods called Matrix Completion (MC) to extract the information coming from a subset of entries of the specialization matrix, to predict remaining entries of that matrix. Such information is encoded by the singular values and singular vectors of a suitable “approximating matrix” constructed automatically by MC. The approach adopted here differs from (truncated) SVD (Hidalgo^2^), because in our framework the specialization matrix is assumed to be only partially observed, in the sense that a given subset of matrix entries, which are provided as inputs to the learning machine in its training phase, is used to predict other portions of the matrix, which are initially artificially hidden to the learning machine. In a second phase, the latter entries are used as a ground truth, for validation and testing purposes. Moreover, we exploit the difficulty in predicting via MC some entries of the specialization matrix to quantify, in an aggregate way, the set of unobservables that make a country more competitive than expected. Such unobservables form the unexplained part of the specialization matrix. For the first time in the literature on economic complexity, a measure of the degree of predictability via MC of the entries of the specialization matrix corresponding to different countries is, then, used to define a novel index of economic complexity for countries (however, our proposed framework can be easily extended to the case of products). We have been inspired by the Total Factor Productivity (TFP) measure in economic growth models. Abramovitz^5^ called TFP “a measure of our ignorance” since it is what is left unexplained by growth in the inputs of the production function. More precisely, our main idea is to adopt MC to infer information about the Relative Comparative Advantage (RCA), or disadvantage, of a country in a given trade category of products. Such information is collected, for each year, in a matrix, $\mathbf{RCA} \in {\mathbb {R}}^{C \times P}$. In formulas, one has

1 $$\begin{aligned} RCA_{c,p}:=\frac{\frac{D_{c,p}}{\sum _{p'=1}^P D_{c,p'}}}{\frac{\sum _{c'=1}^C D_{c',p}}{\sum _{c'=1}^C \sum _{p'=1}^P D_{c',p'}}}\,, \end{aligned}$$

where $D_{c,p}$ is the value in international dollars of the exports of the product *p* by country *c*. In case one among $D_{c,p}$, $D_{c,p'}$, $D_{c',p}$, and $D_{c',p'}$ in Eq. (1) is not available, one gets $RCA_{c,p}=NaN$. In this case, as a pre-processing step, that $RCA_{c,p}$ value can be replaced by 0. In order to extract topological information from the $\mathbf{RCA}$ matrix, it is common in the literature (see, e.g., Sciarra et al.^4^) to consider also the associated incidence matrix $\mathbf{M} \in {\mathbb {R}}^{C \times P}$, whose entries are defined as follows:

2 $$\begin{aligned} M_{c,p} := {\left\{ \begin{array}{ll} 1 \,,&{} \text{ if } RCA_{c,p} \ge 1\,, \\ 0\,, &{} \text{ otherwise }\,. \end{array}\right. } \end{aligned}$$

In the paper, MC is applied several times (starting from different training subsets of suitably discretized RCA values associated with several countries and products, excluding originally *NaN* values) to estimate the expected RCA values of pairs of countries *c* and products *p* that have not been used in the training phase. To fulfill this task, the adopted MC technique is based on a soft-thresholded SVD, which selects each time – via a suitable regularization technique – the subset of most informative singular values and corresponding singular vectors. The work contributes to the literature on economic complexity in three ways: (i) it applies for the first time MC to assess the complexity of countries and to predict the evolution of international trade; (ii) it defines a novel index of economic complexity based on MC; (iii) it builds up a comparison with state-of-the-art indices of economic complexity, revealing a high correlation between the output of GENEPY when it is applied to the original incidence matrix and the false positive rate of a binary classifier derived by the repeated application of MC. The results of our analysis show that MC performs well in estimating the RCA of countries. Supported by the high-quality predictions of MC, we propose a novel Matrix completion iNdex of Economic complexitY (MONEY) for countries, which exploits the accuracy of their RCA predictions derived from the repeated application of MC. Such accuracy is expressed in terms of a suitably weighted Receiver Operating Characteristic (ROC) Area Under the Curve (AUC), one for each country examined. The MONEY index ranks countries according to their degree of predictability, taking into account also the complexity of the products. Specifically, the larger the AUC for a specific country and the larger the average with respect to a subset of the products of that country of the MC performance in estimating the discretized RCA values of country-product pairs, the less complex that country. Using MC to construct the proposed index helps to solve one shortcoming of other economic complexity measures, i.e., the fact that, differently from MC, they take into account only the information coming from the leading eigenvectors. For instance, GENEPY and our proposed application of MC differ in various aspects, also in the particular case in which the specialization matrix has two large singular values followed by other much smaller ones. Indeed, GENEPY takes as input the whole specialization matrix, transforms it into a symmetric matrix, and finds the two largest eigenvalues of the latter matrix. Differently, in the present context, MC is applied several times, for different partitions of the specific specialization matrix into training/validation/test sets. Moreover, for each repetition of MC, the specialization matrix is only partially observed, in such a way that only an approximate SVD can be obtained for it by MC. Moreover, the GENEPY index computed using the MC surrogate incidence matrix reveals interesting discrepancies in terms of economic complexity with respect to the original GENEPY, i.e., the one calculated starting from the incidence matrix associated with the observed **RCA** matrix. Finally, we show that our MC-based classifier has better prediction performance than machine learning methods proposed in Tacchella et al.^6^, which, to the best of our knowledge, is the only work exploiting machine learning to analyze economic complexity and international trade.

## Predicting the economic complexity: a matrix completion approach

In this work, we apply Matrix Completion (MC) techniques to study economic complexity. This class of machine-learning methods has been popularized by the so-called Netflix competition; see the Supplementary material for further details on MC and Hastie et al.^7^, Alfakih et al.^8^, and Cai et al.^9^ for some of its applications. To illustrate the potential of MC in the analysis of economic complexity, in this paper we use MC to estimate the expected Revealed Competitive Advantage (RCA) of countries *c* and products *p*. However, our approach is general and can be applied to different locations (i.e. firms, cities or regions) and for other activities (i.e. patents, scientific production, skills). The specific MC method adopted in the paper consists in completing a partially observed matrix $\mathbf{A} \in {\mathbb {R}}^{C \times P}$ (which is derived from the $\mathbf{RCA}$ matrix in our case, and represents the specialization matrix in our context), by minimizing a suitable trade-off between the reconstruction error of the known portion of that matrix and a penalty term, which penalizes a high nuclear norm of the reconstructed (or completed) matrix. This is formulated via the following optimization problem (Mazumder et al.^10^):

3 $$\begin{aligned} \mathop {\text {minimize}}\limits _{\mathbf{Z} \in {\mathbb {R}}^{C \times P}} \left( \frac{1}{2} \sum _{(c,p) \in \Omega ^{\mathrm{tr}}} \left( A_{c,p}-Z_{c,p} \right) ^2 + \lambda \Vert \mathbf{Z}\Vert _*\right) \,, \end{aligned}$$

where $\Omega ^{\mathrm{tr}}$ is a training subset of pairs of indices (*c*, *p*) corresponding to positions of known entries of the partially observed matrix $\mathbf{A} \in {\mathbb {R}}^{C \times P}$, $\mathbf{Z} \in {\mathbb {R}}^{C \times P}$ is the completed matrix (to be optimized), $\lambda \ge 0$ is a regularization constant (chosen by a suitable validation method), and $\Vert \mathbf{Z}\Vert _*$ is the nuclear norm of the matrix $\mathbf{Z}$, i.e., the sum of all its singular values. A possible state-of-the art iterative algorithm to solve the optimization problem (3) is called Soft Impute (Mazumder et al.^10^). Its main idea consists in replacing iteratively (until convergence) the unobserved elements of the matrix $\mathbf{A}$ with those imputed by a soft-thresholded SVD. The reader is referred to the Supplementary material for further technical details on the optimization problem (3) and on the Soft Impute algorithm.

While MC has already found many applications in several fields (e.g., movie recommendation, sensor engineering, econometrics), to the best of our knowledge, this is the first time it is used to analyze economic complexity. More precisely, we applied MC to construct surrogates of a specialization matrix and to define a novel complexity index, then we compared the obtained results with the ones provided by other state-of-the-art complexity indices.

In the following, we describe our approach of applying MC to the reconstruction of the $\mathbf{RCA}$ matrix for the case in which the products were aggregated at the 4-digits level in the Harmonized System Codes 1992 (HS-1992). In the Supplementary material we provide results at different levels of aggregation of trade (2 and 6 digits). Consistently with the literature (Sciarra et al.^11^), we constructed the matrix $\mathbf{A}$ (one of the inputs to the optimization problem (3)) by discretizing the elements of the $\mathbf{RCA}$ matrix (see the Supplementary material for details on its construction). For the sake of brevity, in the following we describe in detail only the MC application to the definition of a measure of complexity of the locations (i.e., countries in our case). To get a measure of complexity of the activities (i.e., products), it is enough to replace the matrix $\mathbf{A}$ with its transpose (see also the Supplementary material for some related results).

1. For the matrix $\mathbf{A} \in {\mathbb {R}}^{C \times P}$ (where $C=119$ is the number of countries, and $P=1243$ is the number of products), the MC optimization problem (3) was solved $N=1000$ times by the Soft Impute algorithm, based on various choices for the training/validation/test sets (and, as already mentioned, for the regularization parameter $\lambda$).
2. For each such repetition $n=1,\ldots ,N$, the sets above were constructed as follows. First, a (pseudo)random permutation of the rows of $\mathbf{A}$ was generated. Then, a subset $S_n$ of these rows was considered, by including in it the first row in the permutation and the successive $s\% \simeq 25\%$ rows. In this way, the resulting number of elements of the set $S_n$ was $|S_n|=30$. Next, for each row in $S_n$, its elements belonging to all the groups except group “0” (associated with originally *NaN* RCA values) were obscured independently with probability $p_{\mathrm{missing}}=0.3$ (see the Supplementary material for a robustness check of the results of the analysis with respect to the choice of $p_{\mathrm{missing}}$). The (indices of the) remaining entries of the matrix $\mathbf{A}$ (excluding the ones belonging to the group “0”) formed the training set (denoted by $\Omega ^{\text {tr}_n}$). The obscured entries in one of the $|S_n|$ rows (say, row $h \in \{1,\ldots ,|S_n|\}$) formed the test set (denoted by $\Omega ^{\text {test}_{n,h}}$), whereas the obscured entries in the remaining $|S_n|-1$ rows formed the validation set (denoted by $\Omega ^{\text {val}_{n,h}}$).
3. For each repetition *n*, the generation of the validation and test sets from the set $S_n$ was made $|S_n|$ times, each time with a different selection of the row *h* associated with the test set (and, as a consequence, also of the $|S_n|-1$ rows associated with the validation set). Hence, the same training set was associated with $|S_n|$ different pairs of validation and test sets (the number of repetitions $N=1000$ and the percentage $s\% \simeq 25\%$ were selected in order to associate each row with the test set a sufficiently large number of times, with high probability; in particular, with these choices, the average number of times each row was associated with the test set was about 250). In this way, for each choice of $S_n$ and of the regularization parameter $\lambda$, the MC optimization problem (3) was solved once instead of $|S_n|$ times, thus improving the computational efficiency. Finally, by construction, each time there was no overlap between the training, validation, and test sets.
4. To avoid overfitting, for each choice of the training set $\Omega ^{\text {tr}_n}$, the optimization problem (3) was solved for 30 choices $\lambda _k$ for $\lambda$, exponentially distributed as $\lambda _k = 2^{(k-1)/2}$ for $k=1,\dots ,30$. The resulting completed (and post-processed, see the Supplementary material) matrix was indicated as $\mathbf{Z}^{(n)}_{\lambda _k}$. For each $\lambda _k$ and each of the $|S_n|$ selections of the validation sets associated with the same training set, the Root Mean Square Error (RMSE) of matrix reconstruction on that validation set was computed as
  4 $$\begin{aligned} RMSE_{\lambda _k}^{\mathrm{val}_{n,h}}:=\sqrt{\frac{1}{|\Omega ^{\mathrm{val}_{n,h}}|}\sum _{(c,p) \in \Omega ^{\mathrm{val}_{n,h}}} \left( A_{c,p}-Z^{(n)}_{\lambda _k,c,p} \right) ^2}\,, \end{aligned}$$
  then the choice $\lambda _{k^\circ (n,h)}$ minimizing $RMSE_{\lambda _k}^{\mathrm{val}_{n,h}}$ for $k=1,\ldots ,30$ was found (see the Supplementary material for an example of computation of $\lambda _{k^\circ (n,h)}$). Finally, the RMSE of matrix reconstruction on the related test set was computed in correspondence of the so-obtained optimal value $\lambda _{k^\circ (n,h)}$ as
  5 $$\begin{aligned} RMSE_{\lambda _{k^\circ (n,h)}}^{\mathrm{test}_{n,h}}:=\sqrt{\frac{1}{|\Omega ^{\mathrm{test}_{n,h}}|}\sum _{(c,p) \in \Omega ^{\mathrm{test}_{n,h}}} \left( A_{c,p}-Z^{(n)}_{\lambda _{k^\circ (n,h)},c,p} \right) ^2}\,. \end{aligned}$$
5. For each choice of *n* and *h*, the MC predictions contained in the matrix $\mathbf{Z}^{(n)}_{\lambda _{k^\circ (n,h)}}$ were used to build a binary classifier. More precisely, each time an element $A_{c,p}$ of the matrix $\mathbf{A}$ was in the test set, such element was attributed to the class 0 (corresponding to the case $0 \le RCA<1$) when its MC prediction from $\mathbf{Z}^{(n)}_{\lambda _{k^\circ (n,h)}}$ was lower than 0, otherwise it was attributed to the class 1 (corresponding to the case $RCA \ge 1$). Finally, the average classification of the element $A_{c,p}$ (with respect to all the test sets to which that element belonged) was indicated as ${\overline{A}}^{(MC)}_{c,p} \in [0,1]$, whereas its most frequent classification (either 0 or 1) was indicated as ${\hat{A}}^{(MC)}_{c,p}$. A random assignment between 0 and 1 was made to deal with ties. In the (unlikely) case the element $A_{c,p}$ appeared in none of the test sets, both ${\overline{A}}^{(MC)}_{c,p}$ and ${\hat{A}}^{(MC)}_{c,p}$ were chosen to be equal to 0 (due to the choice $p_{\mathrm{missing}}=0.3$, each element $A_{c,p}$ not associated with the group “0” appeared in the test set on average about 75 times; so, the probability that one such element appeared in none of the test sets was negligible).
6. A first MC surrogate $\overline{\mathbf{M}}^{(MC)} \in {\mathbb {R}}^{119 \times 1243}$ of the incidence matrix $\mathbf{M}$ was defined as follows:
  6 $$\begin{aligned} {\overline{M}}^{(MC)}_{c,p}\doteq {\left\{ \begin{array}{ll} 0\,,&{} {\,\,\mathrm if\,\,} RCA_{c,p}=NaN\,, \\ {\overline{A}}^{(MC)}_{c,p}\,,&{} {\,\,\mathrm otherwise\,.} \end{array}\right. } \end{aligned}$$
  Similarly, a second MC surrogate $\hat{\mathbf{M}}^{(MC)} \in {\mathbb {R}}^{119 \times 1243}$ of the incidence matrix $\mathbf{M}$ was defined as follows:
  7 $$\begin{aligned} {\hat{M}}^{(MC)}_{c,p}\doteq {\left\{ \begin{array}{ll} 0\,,&{} {\,\,\mathrm if\,\,} RCA_{c,p}=NaN\,, \\ {\hat{A}}^{(MC)}_{c,p}\,,&{} {\,\,\mathrm otherwise\,.} \end{array}\right. } \end{aligned}$$
7. Finally, in order to assess the prediction capability of the binary classifier associated with MC (see Step 5 above), for each row (country) *c* of $\mathbf{A}$, we also computed the false positive rate ${fpr}_c$ and the false negative rate ${fnr}_c$ as the average classification error frequency, respectively, of the true negative/true positive examples in all the test sets associated with that row (where the “negative class” refers to the class 0 associated with $0 \le RCA<1$, and the “positive class” to the class 1 associated with $RCA \ge 1$).

Concluding, in our application of MC to economic complexity, the MC optimization problem (3) was solved several times by the Soft Impute algorithm, for different choices of the regularization parameter $\lambda$ and of the subset $\Omega ^{\mathrm{{tr}}}$. Then, the two MC surrogates $\overline{\mathbf{M}}^{(MC)}$ and ${\hat{\mathbf{M}}}^{(MC)}$ of the incidence matrix $\mathbf{M}$ were generated. In our successive analysis, such matrices were used in the following way. On one hand, the first MC surrogate $\overline{\mathbf{M}}^{(MC)}$ was exploited to evaluate the performance of MC by changing a suitable threshold from 0 to 1. This allowed to evaluate a set of performance measures that compose our proposed MONEY index (see the next section for details on its definition). On the other hand, the second MC surrogate ${\hat{\mathbf{M}}}^{(MC)}$ was provided as input to the GENEPY algorithm, to construct a counterfactual ${\widehat{\mathrm{GENEPY}}}^{(MC)}$ to be compared with the original $\mathrm{GENEPY}$ index, which is obtained by taking as input to the GENEPY algorithm the original incidence matrix $\mathbf{M}$.

## The matrix completion index of economic complexity (MONEY)

In this section, we introduce our proposed economic complexity index, called Matrix cOmpletion iNdex of Economic complexitY (MONEY), whose construction is based on MC.

**Figure 1:**
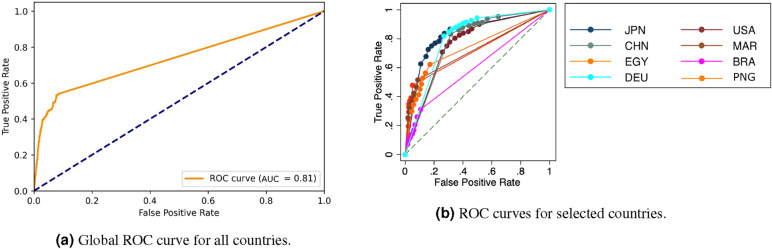
ROC curves constructed starting from the matrix $\overline{\mathbf{M}}^{(MC)}$ for the year 2018. Reference dotted lines passing through the origin with slope 1 are also reported.

The MONEY index is built starting from the matrix $\overline{\mathbf{M}}^{(MC)}$ introduced in the section above. It is based on constructing a binary classifier for each country by combining the corresponding row of $\overline{\mathbf{M}}^{(MC)}$ with a threshold, then assessing the performance of the resulting MC classifications at the level of each country. First, for the binary classifier associated with each country, a Receiver Operating Characteristic (ROC) curve (denoted as $ROC_c$) is constructed, based on a country-dependent threshold. The corresponding Area Under the Curve (AUC) is denoted as $AUC_c$. We remind the reader that, for a binary classifier, the ROC curve expresses the trade-off between fall-out (false positive rate) and sensitivity (true positive rate) of that classifier (both computed on the test set), as a function of its threshold. It is recalled here that the true positive rate is equal to 1 minus the false negative rate. In general, ROC curves closer to the top-left corner indicate a better performance. As a baseline, a random guessing binary classifier is associated with a ROC curve with points lying along the diagonal indicated, e.g., later in Fig. 1a (for which the true positive rate is equal to the false positive rate). The closer a ROC curve to the diagonal in the ROC space, the worse the performance of the associated binary classifier. It is worth reminding the reader that ROC curves do not depend on class frequencies. This makes them useful for evaluating classifiers predicting rare events as in the case of very high RCA values. We also remind the reader that the AUC measures the area of the entire two-dimensional region underneath the entire ROC curve and above the diagonal from (0, 0) to (1, 1). The AUC is exploited in the literature to provide an aggregate measure of performance across all possible classification thresholds. Formally, it represents the probability that a classifier will rank a randomly chosen positive instance higher than a randomly chosen negative one, assuming that “positive” ranks higher than “negative” (Fawcett^12^).

In more detail, for each country *c*, the elements of the *c*-th row of the matrix $\overline{\mathbf{M}}^{(MC)}$ are compared with a threshold to construct the associated binary classifier. The elements belonging to the same row of the original incidence matrix $\mathbf{M}$ are taken as ground truth. The discrimination threshold is varied from 0 to 1, using a step size equal to 0.01. All the elements of $\overline{\mathbf{M}}^{(MC)}$ are used as dataset, except those with the same indices as the originally *NaN* values in the **RCA** matrix. This allows to form a binary classifier for each threshold and for each country. The idea now is to exploit the $AUC_c$ of the binary classifiers associated with the countries in order to provide a measure of complexity of such countries, based on the degree of predictability of the corresponding rows. Specifically, countries with lower $AUC_c$ may be considered as more complex, being harder for MC to predict their RCA entries. The $AUC_c$ alone, however, does not capture the reasons why MC performed poorly (or, vice versa, adequately). As an example, consider the three following hypothetical scenarios. Assume that MC performed poorly on a country *c* by attributing $RCA \ge 1$ to a product *p* when its true RCA was smaller than 1, and assigned correctly a RCA smaller than 1 to all the other countries for the same product *p* (Scenario 1). Consider now the two following similar scenarios for which, for the same product *p* and the same country *c*, MC still performed poorly on *c* by wrongly attributing $RCA \ge 1$ to *p*, and it attributed either correctly (Scenario 2) or incorrectly (Scenario 3) $RCA \ge 1$ to all the other countries for the same *p*. All other things being equal, the country *c* is reasonably more complex in Scenario 1 than in Scenario 2. In fact, while in Scenario 2, MC could have been driven to predict, for country *c*, a RCA of *p* larger than or equal to 1 by the presence of several RCA entries larger than or equal to 1 for the other countries, this is not the case for Scenario 1. Finally, in Scenario 3, it is not possible to conclude that country *c* is more complex than the other countries, since MC is wrongly attributing $RCA \ge 1$ to *p*, for all such countries. Nevertheless, such a scenario is quite unlikely to occur (as it is shown later in the article, MC has typically a quite satisfying prediction capability in its specific application to the discretized **RCA** matrix).

The example above suggests us that, by adopting the $AUC_c$ alone as a complexity measure, country *c* would be classified as equally complex in Scenarios 1, 2 and 3 (assuming the $AUC_c$ being equal in all these cases). In order to correct for this, we propose a refined complexity measure, based on weighting the $AUC_c$ for each country *c*. The rationale of the proposed complexity measure is that not only less predictable countries (according to MC) are more complex, but one should also take into account the product dimension when comparing the MC predictions obtained for different countries, controlling for the quality of each prediction. More precisely, it is proposed to associate a weight $w_{c}$ to each country *c*, which is constructed in such a way that the $AUC_c$’s of countries with an higher share of “rare” false positives are weighted less (since they are less predictable). In more detail, the proposed complexity measure is constructed as follows.

- First, the MC analysis made for the countries is repeated for the products, still referring to the same year. This is obtained simply by replacing at the beginning of the analysis the $\mathbf {RCA}$ matrix with its transpose. Analogously, the matrices $\hat{\mathbf{M}}^{(MC)}$ and $\overline{\mathbf{M}}^{(MC)}$ are replaced by similarly constructed matrices $(\hat{\mathbf{M}}^\top )^{(MC)}$ and $(\overline{\mathbf{M}}^\top )^{(MC)}$. In particular, each element of the latter matrix represents the average MC prediction for the corresponding product-country pair.
- Then, for a given set *T* of thresholds, a threshold $t { \in T}$ is applied to the elements of the matrix $(\overline{\mathbf{M}}^\top )^{(MC)}$. For each value of that threshold, one constructs a matrix $\left( \overline{\mathbf{M}}_t^\top \right) ^{(MC)} \in \{0,1\}^{P \times C}$, being each entry of it equal to 1 whenever the corresponding element in the matrix $(\overline{\mathbf{M}}^\top )^{(MC)}$ is higher than or equal to *t*, otherwise being it equal to 0.
- At this point, for each product *p* and each threshold *t*, one computes the quantity
  8 $$\begin{aligned} ftot_{p,t}:=fpr_{p,t} \times \frac{N_p}{N_p + P_p}\,, \end{aligned}$$
  being $fpr_{p,t}$ the false positive ratio for the classifications associated with that product (determined by the comparison between $\left( \overline{\mathbf{M}}_t^\top \right) ^{(MC)}$ and $\mathbf{M}^\top$, restricted to the entries associated with that product) and $\frac{N_p}{N_p + P_p}$ the proportion of entries with true $RCA <1$ with respect to all the entries associated with that product (i.e., 119).
- Moreover, the average
  9 $$\begin{aligned} { {\overline{ftot}}_{p}:=\frac{\sum _{t \in T} ftot_{p,t}}{|T|}} \end{aligned}$$
  of $ftot_{p,t}$ with respect to $t { \in T}$ is computed.
- Then, for each country *c*, one computes both $AUC_c$ and a weight $w_c$, which is defined as follows:
  10 $$\begin{aligned} w_c:=\frac{\sum _{p=1}^P ({\hat{M}}^\top )^{(MC)}_{p,c} \times {\overline{ftot}}_p}{\sum _{p=1}^P ({\hat{M}}^\top )^{(MC)}_{p,c}}\,. \end{aligned}$$
  In other words, for each country *c*, the weight $w_c$ is the average of ${\overline{ftot}}_{p}$ with respect to all the products *p* for which one predicts $RCA\ge 1$ through the MC surrogate incidence matrix $(\hat{\mathbf{M}}^\top )^{(MC)}$.
- Finally, the MONEY index for each country *c* is computed as:
  11 $$\begin{aligned} MONEY_c:=1-w_{c} \times AUC_{c}\,. \end{aligned}$$

## Results

### Global performance of matrix completion

In this section we report some summary statistics of the prediction performance of MC. Likewise in the section above, the matrix $\overline{\mathbf{M}}^{(MC)}$ was combined with a threshold to construct a binary classifier (in this case, however, differently from that section, the threshold did not depend on the country). The discrimination threshold was varied from 0 to 1, using a step size equal to 0.01. All the elements of $\overline{\mathbf{M}}^{(MC)}$ were used as dataset, except the ones having the same indices as the originally *NaN* values in the **RCA** matrix. The ground truth was provided by the corresponding elements of the original incidence matrix $\mathbf{M}$. Figure 1a shows the resulting ROC curve. The global AUC for the matrix $\overline{\mathbf{M}}^{(MC)}$ when compared to the real-world matrix of year 2018 is 0.81. Similarly, $ROC_c$ curves for selected countries are displayed in Fig. 1b.

As it is evident from Fig. 1, MC performed quite well on average both globally and for developed countries such as Japan, United States and Germany. Its performance was poorer (though still above the baseline) for countries that either provided less information on their trade flows or whose trade flows were extremely volatile (i.e., they alternated between products with extremely high RCA values and products with very low RCA values). Specifically, ${fnr}_c$ was higher for the latter countries. Nonetheless, the average performance of MC over all the countries was high as depicted by the AUC reported in Fig. 1a. As a further check, since the positive and negative labels were unbalanced in the original dataset (specifically, entries with $RCA<1$ represented almost the 70% of the entire dataset), we also computed the Balanced Accuracy (BACC) index, which turned out to be 0.75 (with a threshold on $\overline{\mathbf{M}}^{(MC)}$ equal to 0.5). We recall that the BACC is a performance metric designed for binary classifiers in the case of unbalanced datasets. It is calculated as the average of the proportion of correctly classified elements of each class individually, and ranges from 0 (low balanced accuracy) to 1 (maximum balanced accuracy). Formally, it is equal to $\left( {tpr} + {tnr} \right) / 2$, where *t* stands for “true”. An alternative index used for unbalanced datasets is the F1 score, which is defined as $F1=\frac{TP}{TP+\frac{1}{2}(FP+FN)}$, where *TP*, *FP* and *FN* represent, respectively, the number of true positives, false positives, and false negatives on the test set. The F1 score turns out to be equal to 0.73 for ${\hat{\mathbf{M}}}^{(MC)}$ (i.e., at the 0.5 threshold level for ${\overline{\mathbf{M}}}^{(MC)}$), whereas the best F1 score (obtained by optimizing the threshold on ${\overline{\mathbf{M}}}^{(MC)}$) is 0.74. In the Supplementary material we repeated the analysis at a more refined level of aggregation of world trade (HS-6 level). At the HS-6 level the global AUC reduced to 0.72 and the best F1 score of MC when applied at the HS-6 level turned out to be 0.73. This is a good performance considering that no other feature of products and countries was used for predictions but various subsets of elements of the matrix $\mathbf{A}$, given as inputs to MC.

**Figure 2:**
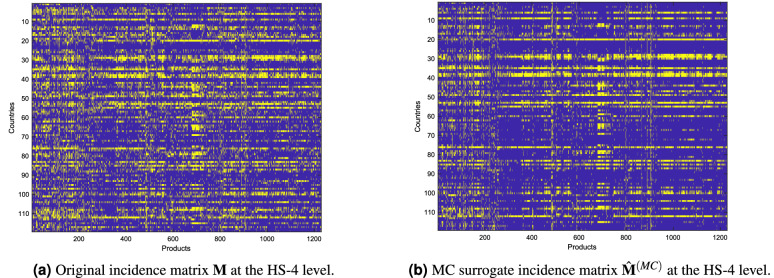
Similarity between the original incidence matrix $\mathbf{M}$ and the MC surrogate incidence matrix $\hat{\mathbf{M}}^{(MC)}$ for the year 2018 at the HS-4 level, confirming the good MC prediction performance at a global level.

Figure 2 displays the original incidence matrix $\mathbf{M}$ as compared to the MC surrogate incidence matrix $\hat{\mathbf{M}}^{(MC)}$ obtained at the HS-4 level of product aggregation (in the figure, blue stands for 0 and yellow for 1). The two matrices display similar but not identical entries. On one hand, their similarity confirms the good MC prediction performance at a global level. On the other hand, their differences could be attributed to the high complexity of specific country/product pairs being predicted. In other words, there may be a discrepancy between the actual RCA value of a country/product pair and its potential RCA value, predicted by MC on the basis of similar country/product pairs.

### The MONEY index

Results in the previous section highlight that MC reaches a good performance in predicting the RCA values of country-product pairs. However, some countries have more false positive predictions than others.

**Figure 3:**
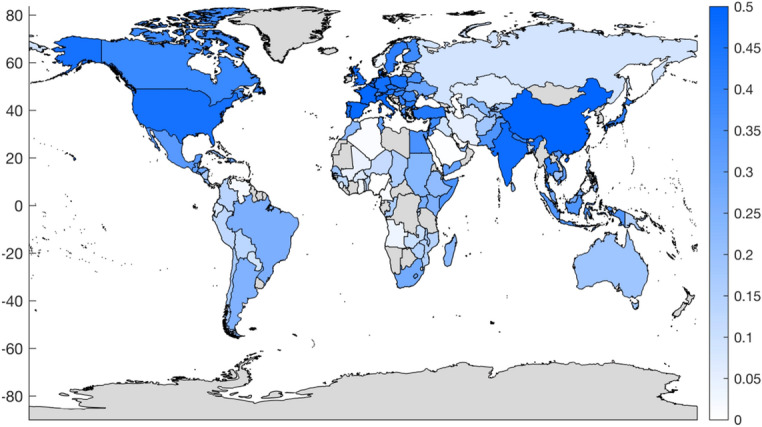
False positive rate ${fpr}_c$, reported proportionally to the shade of blue for the year 2018 and the product aggregation level HS-4. The map was generated using the MATLAB 2012b package borders, available for free (upon registration) at the following hyperlink: https://it.mathworks.com/matlabcentral/fileexchange/50390-borders.

Figure 3 displays the false positive rate ${fpr}_c$ for each country considered in the analysis. Surprisingly, the ranking of countries by false positive rate turns out to be quite similar to the one generated by the GENEPY economic complexity index (Kendall rank correlation coefficient $\tau _k=0.75$). This is reassuring since the key insight of the MONEY index is that the activities of more complex countries are more difficult to predict, as confirmed by the strong correlation between the false positive rate of MC and GENEPY.

**Figure 4:**
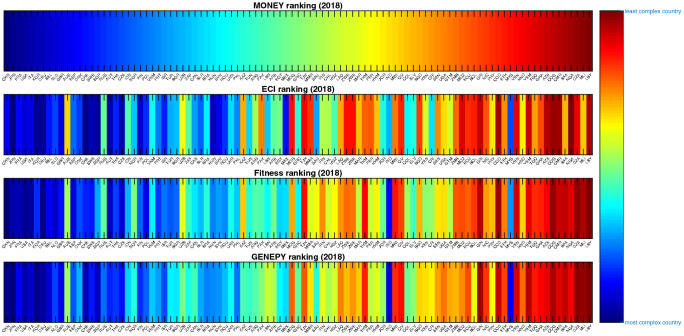
Comparison table for various country complexity indices in 2018 (HS-4 level).

This section reports also the ranking of countries by economic complexity as expressed by the MONEY index. Results are compared to other popular measures of economic complexity (see Fig. 4): ECI from Hidalgo and Hausmann^1^, Fitness from Tacchella et al.^13^, and GENEPY from Sciarra et al.^4^. Overall, the rankings are similar, with some remarkable differences for the MONEY ranking. Australia ranks much higher (and in the top positions) according to MONEY than all other indices. In the bottom part of the MONEY ranking, we find Malaysia, which ranks higher based on the other economic complexity measures.

Despite some relevant differences, economic complexity indices turn out to be quite correlated. Table 1 reports the values of the Kendall rank correlation coefficient $\tau _k$ when comparing the rankings (world trade at the HS-4 level) provided by ECI, Fitness, GENEPY, MONEY, and GDP per capita Purchasing Power Parity (PPP) for the year 2018. The level of correlation of MONEY with the other economic complexity indices and with GDP per capita, PPP is similar to the one of ECI, Fitness, and GENEPY.

**Table 1:** Kendall rank correlation coefficient $\tau _k$ for economic complexity indices (ECI, Fitness, GENEPY and MONEY) and the GDP per capita, PPP. World trade at the HS-4 level.

		**Economic complexity and GDP per capita, PPP**				
Year	Index	ECI	Fitness	GENEPY	MONEY	GDP per capita, PPP
2018	ECI	1	0.75	0.74	0.61	0.59
	Fitness	.	1	0.82	0.69	0.55
	GENEPY	.	.	1	0.73	0.57
	MONEY	.	.	.	1	0.57

**Figure 5:**
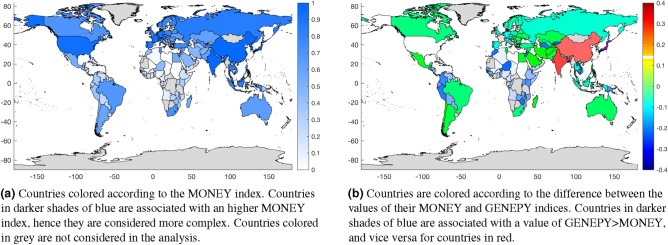
MONEY index for the year 2018 at the HS-4 level of aggregation and the difference with to the corresponding values of the GENEPY index for the same year and the same level of the product classification. The maps were generated using the MATLAB 2012b package borders, available for free (upon registration) at the following hyperlink: https://it.mathworks.com/matlabcentral/fileexchange/50390-borders.

In the following we take a closer look to the differences between MONEY and GENEPY (see Fig. 5a). Both indices are normalized to the interval [0, 1]. In Figure 5a, countries are colored according to their MONEY values, which are proportional to the shade of blue: the color map ranges from the least complex countries (colored in white) to the most complex ones (colored in dark blue). Figure 5b shows the difference between the normalized values of MONEY and GENEPY. A different color map is used in Fig. 5b, due to its different meaning with respect to Fig. 5a. Since developed countries tend to have similar export baskets (Sciarra et al.^4^), the predictability of their RCA values via MC might be higher, hence reducing their MONEY index to some extent.

Both GENEPY and MONEY indices arise from the attempt to reconstruct (in a different way for each method) a matrix related to trade flows. In the case of GENEPY, the matrix is a proximity matrix $\mathbf{N}$ derived from the incidence matrix $\mathbf{M}$ (see the Supplementary material for the definition of the matrix $\mathbf{N}$), and its reconstruction is obtained as a nonlinear least-square estimate based on the components of the first two (normalized) eigenvectors of that matrix. Then, a successive evaluation on how the quality of the estimate changes by dropping specific components of such eigenvectors (the ones associated with a given country) is made. In our case, the matrix $\mathbf{A}$ is obtained as a discretization of the $\mathbf{RCA}$ matrix. Then, MC is applied several times to the matrix $\mathbf{A}$ to reconstruct a portion of that matrix which has been obscured, in the attempt to uncover a “latent” similarity between countries, which can be useful for the prediction of RCA entries. Another difference is that the matrix reconstruction on which GENEPY is based relies only on two eigenvectors of $\mathbf{N}$, whereas our method, being also based on MC, exploits a typically much larger number of left-singular/right-singular vectors to build the reconstructed matrix, for each application of MC. The choice of the number of such pairs is made automatically by the adopted validation procedure. Further comparative results are available in the Supplementary material for different years and aggregation levels.

### Differences in the GENEPY indices based on the original incidence matrix $\mathbf{M}$ and on $\hat{\mathbf{M}}^{(MC)}$

Another potential use of MC is to predict the missing values in the trade dataset, in a similar way as in Metulini et al.^14^. Missing values amount to around $25\%$ of the entries of the $\mathbf{M}$ matrix. This implies using, for the originally *NaN* entries of that matrix, the corresponding values of their most frequent classifications derived from MC. To test how reliable this approach is, in this section, we compare the values of the GENEPY index with the value of GENEPY computed by using the surrogate matrix ${\hat{\mathbf{M}}}^{(MC)}$, that is to say ${\widehat{\mathrm{GENEPY}}}^{(MC)}$.

To quantify the correlation between the GENEPY rankings computed based on $\mathbf{M}$ and $\hat{\mathbf{M}}^{(MC)}$, respectively, we evaluated their Kendall rank correlation coefficient $\tau _k$. The statistical test produced $\tau _k \simeq 0.8$ with a *p*-value near 0, rejecting significantly the null hypothesis of independence between GENEPY and ${\widehat{\mathrm{GENEPY}}}^{(MC)}$. With a few exceptions (China, France, Italy, UK and Germany) the more complex the country according to GENEPY, the higher the difference between GENEPY and ${\widehat{\mathrm{GENEPY}}}^{(MC)}$. Despite machine learning opens to new possible solutions for the imputation of missing values, more progress is needed to refine the performance of MC for missing imputation, possibly combining it with other machine learning techniques (Metulini et al.^14^).

## Discussion

Machine learning has an enormous potential to enhance the quality of the prediction of economic growth and competitiveness (Longo et al.^15^). In the present work, we applied Matrix Completion (MC) to investigate the economic complexity of countries in various ways . First, we assessed the high accuracy of the MC predictions, when MC was applied to reconstruct the Revealed Comparative Advantage (RCA) matrix, which is at the basis of the construction of several existing economic complexity indices (see the Supplementary material). Then, we proposed the Matrix cOmpletion iNdex of Economic complexitY (MONEY), based on the degree of predictability of the RCA entries associated with different countries. The MONEY index is based on the idea that complex economic systems are more difficult to predict. As an additional contribution, we compare MC with recently-developed economic complexity indices, to assess the expected economic complexity of countries. As an example, in the work, MC was exploited to infer the expected discretized RCA of a country *c*  for a product *p*. The MC technique employed is based on a soft-thresholded SVD. This, combined with the MC validation phase, allows to select automatically a suitable number of singular vectors to be used to reconstruct the $\mathbf{RCA}$ matrix. Differently from previous economic complexity indices, the information extracted by MC is not restricted to the first two singular vectors, but a suitable number of singular vectors is selected to optimize the out-of-sample prediction performance.

Our results highlighted a good performance of MC in discerning country-product pairs with RCA values greater than or equal to the critical threshold of 1, denoting the expected competitiveness of country *c* in exporting product *p*. The outcomes were summarized by reporting the global ROC curve and comparing the heat-map of the true incidence matrix $\mathbf{M}$ and the one of its MC surrogate matrix $\hat{\mathbf{M}}^{(MC)}$, which was obtained from various applications of MC. Motivated by the high MC accuracy, we developed the MONEY index taking into account both the predictive performance of MC for each country (as measured by its $AUC_c$) and the product dimension. In other words, when constructing that index, each $AUC_c$ was weighted by the average of the ${\overline{ftot}}_p$’s with respect to a subset of products associated with the specific country. The MONEY index can be used as a measure of economic complexity to predict the future growth potential of countries. Moreover, MC can help to deal with missing values, when the missingness pattern is not random. More in general, future research is needed to further increase the quality of the predictions of machine learning methods when applied to economic complexity.

## Supplementary Information

Supplementary Information.

## Data Availability

The RCA values used in our analysis come from the CEPII - BACI dataset, which is freely distributed under the Etalab Open License 2.0, and can be retrieved at the following hyperlink: http://www.cepii.fr/cepii/en/bdd_modele/bdd.asp.
