# Supplementary material for: A machine learning approach to economic complexity based on matrix completion
Source: Sci Rep. 2022 Jun 10;12:9639. doi: 10.1038/s41598-022-13206-0 (PMC9187690; doi:10.1038/s41598-022-13206-0)
Supplement: Supplementary file 1 — Supplementary Information. [file 41598_2022_13206_MOESM1_ESM.pdf]

Supplementary material

Background material

Matrix completion via nuclear norm regularization of the reconstruction error

Given a subset of observed entries of a matrix $\mathbf{A} \in \mathbb{R}^{C \times P}$, Matrix Completion (MC) works by finding a suitable low-rank approximation (say, with rank R) of $\mathbf{A}$, by assuming the following model:

$$\mathbf{A} = \mathbf{C}\mathbf{G}^\top + \mathbf{W}, \tag{S1}$$

where $\mathbf{C} \in \mathbb{R}^{C \times R}$, $\mathbf{G} \in \mathbb{R}^{P \times R}$, whereas $\mathbf{W} \in \mathbb{R}^{C \times P}$ is a matrix of modeling errors. The rank-R approximating matrix $\mathbf{C}\mathbf{G}^\top$ is found by solving a suitable optimization problem (provided by Eq. (3), in the case of the present article). Eq. (S1) can be written element-wise as $A_{c,p} = \sum_{r=1}^{R} C_{c,r} G_{p,r} + W_{c,p}$. A common interpretation of this equation is as follows (see, e.g., the application of MC to collaborative filtering for movie ratings, as in Hastie et al., 2015[3]). The number $C_{c,r}$ can be interpreted as the degree of membership of row c of matrix $\mathbf{A}$ to some "latent" cluster r (for a total of R such clusters), and $G_{p,r}$ as the prediction of an element in column p of matrix $\mathbf{A}$, conditioned on its row c belonging to cluster r. It is worth mentioning that such an interpretation holds regardless of the signs of the elements $C_{c,r}$ and $G_{p,r}$. As an example, in the case of collaborative filtering for movie ratings, c denotes a specific person, p a specific movie, whereas r may be interpreted as a specific movie genre.

In this work, MC is formulated via the optimization problem (3). The objective function of this optimization problem is the sum of two terms: the first one refers to the reconstruction error of the known portion of the matrix, whereas the second one is a regularization term, which biases the reconstructed matrix to have a small nuclear norm. The regularization constant λ controls the trade-off between fitting the known entries of the matrix $\mathbf{A}$ and achieving a small nuclear norm. The latter requirement is often related to getting a low rank of the obtained optimal matrix $\mathbf{Z}_\lambda^\circ$, which follows by geometric arguments similar to the ones typically adopted to justify how the classical LASSO (Least Absolute Shrinkage and Selection Operator) penalty term achieves effective feature selection in linear regression (Tibshirani, 1996[13]).

The MC optimization problem (3) can be also written as

$$\underset{\mathbf{Z} \in \mathbb{R}^{C \times P}}{\text{minimize}} \left(\frac{1}{2} \| \mathbf{P}_{\Omega^{\text{tr}}}(\mathbf{A}) - \mathbf{P}_{\Omega^{\text{tr}}}(\mathbf{Z}) \|_F^2 + \lambda \|\mathbf{Z}\|_* \right), \tag{S2}$$

where, for a matrix $\mathbf{Y} \in \mathbb{R}^{C \times P}$, $(P_{\Omega^{\text{tr}}}(\mathbf{Y}))_{c,p} := Y_{c,p}$ if $(c,p) \in \Omega^{\text{tr}}$, otherwise it is equal to 0. Here, $P_{\Omega^{\text{tr}}}(\mathbf{Y})$ represents the projection of $\mathbf{Y}$ onto the set of positions of observed entries of the matrix $\mathbf{A}$, and $\|\mathbf{Y}\|_F$ denotes the Frobenius norm of $\mathbf{Y}$ (i.e., the square root of the summation of squares of all its entries).

The MC optimization problem (S2) can be solved by applying the following Algorithm 1, named Soft Impute in Mazumder et al. (2010)[8] (compared to the original version, here we have included a maximal number of iterations N^{it}, which can be helpful to reduce the computational effort when one has to run the algorithm multiple times, e.g., for several choices of the training set Ω^{tr} and of the regularization constant λ, as in the present work):

Algorithm 1: Soft Impute (Mazumder et al., 2010[8])

Input: Partially observed matrix $\mathbf{P}_{\Omega^{\text{tr}}}(\mathbf{A})$, regularization constant $\lambda \geq 0$, tolerance $\varepsilon \geq 0$, maximal number of iterations N^{it}

Output: Completed matrix $\mathbf{Z}_\lambda \in \mathbb{R}^{C \times P}$

1. Initialize $\mathbf{Z}$ as $\mathbf{Z}^{\text{old}} = \mathbf{0} \in \mathbb{R}^{C \times P}$
2. Repeat for at most N^{it} iterations:
   (a) Set $\mathbf{Z}^{\text{new}} \leftarrow \mathbf{S}_\lambda \left(\mathbf{P}_{\Omega^{\text{tr}}}(\mathbf{A}) + \mathbf{P}_{\Omega^{\text{tr}}}^{\perp}(\mathbf{Z}^{\text{old}}) \right)$
   (b) If $\frac{\|\mathbf{Z}^{\text{new}} - \mathbf{Z}^{\text{old}}\|_F^2}{\|\mathbf{Z}^{\text{old}}\|_F^2} < \varepsilon$, exit
   (c) Set $\mathbf{Z}^{\text{old}} \leftarrow \mathbf{Z}^{\text{new}}$
3. Set $\mathbf{Z}_\lambda \leftarrow \mathbf{Z}^{\text{new}}$

In Algorithm 1, for a matrix $\mathbf{Y} \in \mathbb{R}^{C \times P}$, $\mathbf{P}_{\Omega^{\text{tr}}}^{\perp}(\mathbf{Y})$ represents the projection of $\mathbf{Y}$ onto the complement of Ω^{tr}, whereas $\mathbf{S}_\lambda(\mathbf{Y}) := \mathbf{U}\mathbf{\Sigma}_\lambda \mathbf{V}^\top$, being $\mathbf{Y} = \mathbf{U}\mathbf{\Sigma}\mathbf{V}^\top$ (with $\mathbf{\Sigma} = \text{diag}[\sigma_1, \ldots, \sigma_R]$) the singular value decomposition of $\mathbf{Y}$, and $\mathbf{\Sigma}_\lambda := \text{diag}[(\sigma_1 - \lambda)_+, \ldots, (\sigma_R - \lambda)_+]$, with $t_+ := \max(t, 0)$.

It is worth mentioning that a particularly efficient implementation of the operator $\mathbf{S}_\lambda(\cdot)$ is possible (by means of the MATLAB function `svt.m`, see Li and Zhou, 2017[6]), which is based on the determination of only the singular values σ_i of $\mathbf{Y}$ that are

higher than λ, and of their corresponding left-singular vectors $\mathbf{u}_i$ and right-singular vectors $\mathbf{v}_i$. Indeed, all the other singular values of $\mathbf{Y}$ are annihilated in $\boldsymbol{\Sigma}_\lambda$. A final remark has to be made about the trade-off between prediction capability and biasedness of MC. Biasedness in MC depends, among others issues, on the way the selection of unobserved entries is made (see, e.g., Foucart et al., 2017[1] and Ma and Chen, 2019[7]). In the specific case of our application of MC to the discretized **RCA** matrix, only entries belonging to a suitable subset of rows of the matrix $\mathbf{A}$ are obscured. For some MC algorithms, de-biasing is possible (Foucart et al., 2017[1]), and can even improve prediction capability. Nevertheless, in general biasedness can be beneficial to prediction capability, due to the well-known trade-off between bias and variance (Hastie et al., 2009[2]). In the particular case of MC achieved via the Soft Impute algorithm, biasedness can be ascribed also to the presence of the regularization constant λ (indeed, for both $\lambda \to 0^+$ and $\lambda \to +\infty$, the predictions of the optimal solution to the optimization problem (S2) tend to 0 for the unobserved entries), and to the fact that the Soft Impute algorithm is initialized by a matrix with all entries equal to 0, and terminated at most after a given number of iterations.

Technical details on the construction of the matrix $\mathbf{A}$ and on the the application of the Soft Impute algorithm

This subsection details the construction of the matrix $\mathbf{A}$ for our specific problem. As a first step, we removed from the **RCA** matrix its rows associated with countries having less than 5 million inhabitants, as they presented, at the HS-4 level, a quite high percentage of originally *NaN* RCA values (more than 60%), for which it was not possible to check the quality of the MC prediction. Then, the remaining entries of the **RCA** matrix were encoded into 9 groups according to increasing percentiles in the distribution of RCA values. This pre-processing step was done in order to make the elements of the resulting matrix $\mathbf{A} \in \mathbb{R}^{119\times1243}$ of the same order of magnitude. As already reported in this Supplementary material, MC is biased towards low absolute values. To take this into account, we constructed groups that were symmetrically distributed around zero, as the final goal was to discriminate between RCA values respectively lower than 1, and larger than or equal to 1. In particular, we defined 4 negative groups ("-4", "-3", "-2", "-1"), representing the case $0 \leq RCA < 1$ (with the group "-4" being the one associated with the lowest values in the RCA distribution) and 4 positive groups ("1", "2", "3", "4"), representing the case $RCA \geq 1$ (with the group "4" being the one associated with the highest values in the RCA distribution). Originally *NaN* RCA values were included in the remaining group "0". In our application of MC, the elements in this group "0" were included neither in the training set, nor in the validation/test set, since no ground truth was available for them.

For computational efficiency reasons, we combined the original MATLAB implementation of Soft Impute (Mazumder et al., 2010[8]) with the MATLAB function `svt.m` (Li and Zhou, 2017[6]). The tolerance of the algorithm was chosen as $\varepsilon = 10^{-9}$. Its number of iterations was set to $N^{\mathrm{it}} = 1500$. The regularization parameter λ was sampled 30 times uniformly on the closed interval $[-1, 15]$ in a logarithmic scale with base 2. A post-processing step was included in MC, thresholding to -4 any element (when present) whose MC reconstruction was lower than -4, and to 4 any element (when present) whose MC reconstruction was higher than 4.

Generalized economic complexity index and related economic complexity indices

The GENeralised Economic comPlexitY (GENEPY) index is a recently-introduced economic complexity index (Sciarra et al., 2020[10]), which can be applied to assess the complexity of both countries and products. It is based on a multidimensional representation of their complexity, which makes it possible to combine, in a single index, the different features of some previously-developed one-dimensional economic complexity indices: the Fitness (F) for countries and Quality (Q) for products, both computed by the Fitness and Complexity (FC) algorithm (Tacchella et al., 2012[11]), and the Economic Complexity Index (ECI) for countries and Product Complexity Index (PCI) for products, both obtained by the earlier Method of Reflections (MR), see Hidalgo and Hausmann (2009)[5]. Each of the latter methods is typically able, indeed, to capture only a specific aspect of economic complexity: for instance, when applied to countries, FC is mainly related to the degree of diversification of the export basket of each country, while MR essentially captures the similarities in the export baskets of the different countries (Sciarra et al., 2020[10]).

The GENEPY index arises from the first two (normalized) eigenvectors (with the eigenvalues ordered in a weakly decreasing way) of a suitable symmetric proximity matrix, which is derived from the incidence matrix $\mathbf{M} \in \mathbb{R}^{C\times P}$ obtained by thresholding and binarizing the matrix of Revealed Comparative Advantage (RCA) values. The two eigenvectors capture, respectively, information obtained by the FC method and the MR one.

The GENEPY index for countries is obtained in the following way (a similar construction holds for the GENEPY index for products).

1. First, for a specific year, the matrix $\mathbf{RCA} \in \mathbb{R}^{C\times P}$ of RCA values in that year is determined, and the incidence matrix $\mathbf{M} \in \mathbb{R}^{C\times P}$ is generated from it (see the Introduction for details). Then, a weighted version $\mathbf{W} \in \mathbb{R}^{C\times P}$ of the incidence matrix $\mathbf{M}$ is considered. Its generic element is defined as $W_{c,p} := \frac{M_{c,p}}{k_c k'_p}$, where $k_c := \sum_{p=1}^{P} M_{c,p}$ is the degree of the country c in the graph represented by the incidence matrix $\mathbf{M}$, and $k'_p := \sum_{c=1}^{C} \frac{M_{c,p}}{k_c}$ represents the degree of the product p corrected by how easily that product is found within the subnetwork of countries.

2. The matrix $\mathbf{N} \in \mathbb{R}^{\mathbf{C}\times\mathbf{C}}$ is constructed, whose elements N_{c,c^*} are defined as follows:

$$N_{c,c^*} := \begin{cases} \sum_{p=1}^{P} W_{c,p} W_{c^*,p}, & \text{if } c \neq c^*, \\ 0, & \text{otherwise}. \end{cases} \tag{S3}$$

   Due to the weighting involved in the construction of the matrix $\mathbf{W}$, the resulting matrix $\mathbf{N}$ is symmetric. Each entry N_{c,c^*} of $\mathbf{N}$ represents the proximity of the two corresponding countries c and c^*.
3. The (normalized) eigenvectors $\mathbf{x}_1, \mathbf{x}_2 \in \mathbb{R}^C$ associated with the two largest eigenvalues $\lambda_1 \geq \lambda_2 \geq 0$ of $\mathbf{N}$ are determined. Their components are denoted as $x_{c,1}$ and $x_{c,2}$, respectively, for $c = 1, \ldots, C$.
4. Then, the GENEPY index of country c for the specific year is defined as follows:

$$GENEPY_c := \left(\sum_{i=1}^{2} \lambda_i x_{c,i}^2 \right)^2 + 2 \sum_{i=1}^{2} \lambda_i^2 x_{c,i}^2 . \tag{S4}$$

   The specific nonlinear transformation from $x_{c,1}$ and $x_{c,2}$ to $GENEPY_c$, which is used in Eq. (S4), can be justified by rigorous statistical arguments, based on the use of the two (normalized) eigenvectors $\mathbf{x}_1$ and $\mathbf{x}_2$ to get a nonlinear least-square estimate of the matrix $\mathbf{N}$, and on the evaluation of how relevant $x_{c,i}$ and $x_{c,2}$ are to obtain that estimate (see Sciarra et al., 2018[9] and Sciarra et al., 2020[10]).

It is worth mentioning the qualitative difference between the GENEPY index and the ones determined by the FC and MR methods, considering again the case in which they are all applied to countries.

- The GENEPY index is highly related to a linearized version of the F index computed by the FC method (Sciarra et al., 2020[10]), in which one searches for the (normalized) eigenvector associated with the largest eigenvalue of a slightly different matrix $\mathbf{N}_F \in \mathbb{R}^{C\times C}$ than the matrix $\mathbf{N}$. The specific matrix $\mathbf{N}_F$ is written as $\mathbf{N}_F := \mathbf{W}\mathbf{W}^\top$, where $\mathbf{W}$ is the same weighted incidence matrix considered in the context of the GENEPY index. The difference with respect to the case of the matrix $\mathbf{N}$ defined in Eq. (S3) is that its diagonal entries are not set to 0 (in Eq. (S3), such a choice of the diagonal entries is done in order to make the resulting $\mathbf{N}$ be a proximity matrix).
- The ECI index, computed by MR, is based on searching for the (normalized) eigenvector associated with the second-largest eigenvalue of a slightly different matrix $\mathbf{N}_{ECI} \in \mathbb{R}^{C\times C}$ than the matrix $\mathbf{N}$ considered by GENEPY. The specific matrix $\mathbf{N}_{ECI}$ is written as $\mathbf{N}_{ECI} := \mathbf{W}_{ECI}\mathbf{W}_{ECI}^\top$, where the elements of $\mathbf{W}_{ECI} \in \mathbb{R}^{C\times P}$ are defined as $W_{ECI,c,p} := \frac{M_{c,p}}{k_c k_p}$, being $k_p := \sum_{c=1}^{C} M_{c,p}$ the degree of the product p in the graph represented by the incidence matrix $\mathbf{M}$. The second-largest eigenvalue of $\mathbf{N}_{ECI}$ is considered, instead of its first-largest one, as one can show that the (normalized) eigevector associated with the latter is non-informative, for the specific matrix $\mathbf{N}_{ECI}$.

Similar comments hold for the case of the FC and MR methods when they are applied to products (obtaining, respectively, the Q index and the PCI index).

The MATLAB code to compute the GENEPY index was found at the hyperlink https://zenodo.org/record/3876721#.Ym2vwOhBzIU. Concerning the Fitness (F) index proposed by Tacchella et al. (2012)[11], once established that its authors considered the HS-4 product level, we were able to recover the rankings of countries from 2005 to 2015 in the website of Prof. Pietronero (http://www.lucianopietronero.it/), who provides a "Fitness rankings" file. We thank Giulio Virginio Clemente for providing the values of Fitness for 2018. For the ECI, it was possible to recover the rankings of countries for the years 2005, 2014 and 2018 at the HS-4 product level, by exploiting the well-known Atlas of Economic Complexity (https://atlas.cid.harvard.edu/) from Harvard, which explicitly computes ECI and PCI. For a further check, we computed these rankings also starting from the dataset at our disposal (BACI from CEPII, see http://www.cepii.fr/cepii/en/bdd_modele/bdd.asp) and applying the *ecomplexity* package in Python. The latter rankings were in line with the ones provided by the Atlas of Economic Complexity.

Determination of the optimal value of the regularization parameter λ, and its comparison with a different choice of λ

Supplementary Fig. 1 shows, for a specific simulation at the HS-4 level and for the year 2018, the behavior of the RMSE on the training, validation, and test sets (analogous results were obtained for the other simulations). The figure also shows how the optimal value of λ was obtained, by minimizing the RMSE on the validation set. In this particular simulation, in correspondence of the optimal choice of λ, MC and the post-processing step produced 82 largest singular values (where, in the specific case, "largest" means larger than or equal to 10% of the value assumed by the maximum singular value). The figure also highlights another choice of λ, for which the number of largest singular values obtained was only 2. In the latter case, it is evident from the figure that the corresponding RMSEs on the validation and test sets were much larger with respect to the ones associated with the optimal choice of λ.

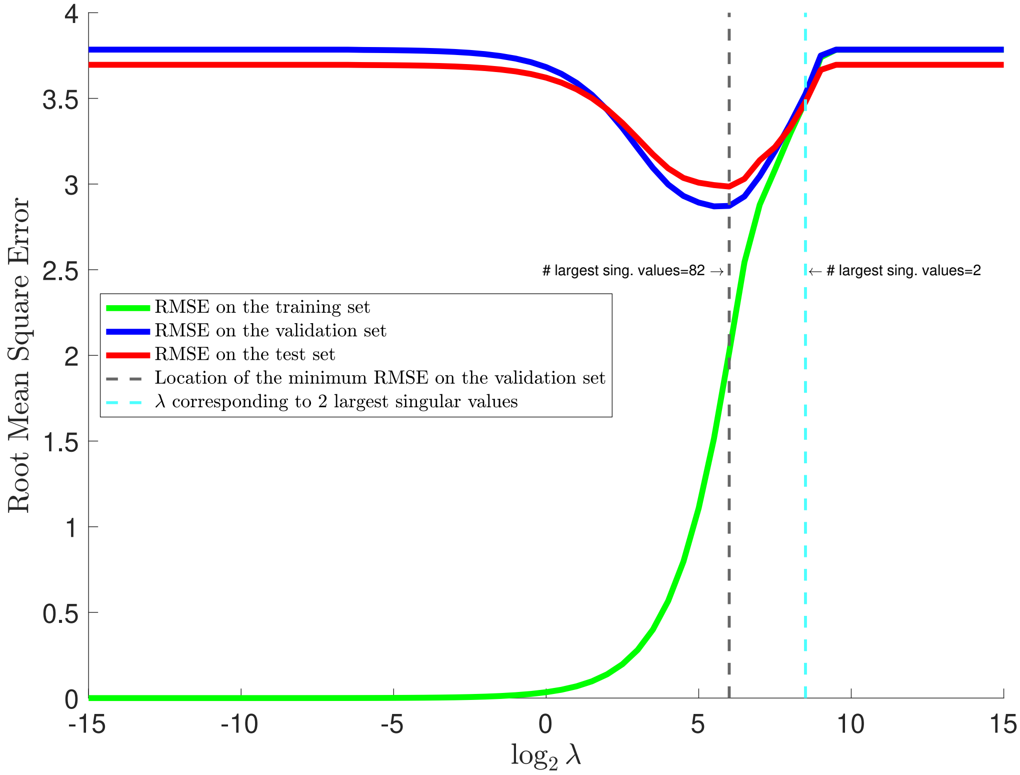

Supplementary Figure 1. RMSE as a function of $\log_2(\lambda)$, for a specific choice of the training/validation/test sets (HS-4 level; year: 2018).

Robustness check of the results of the analysis with respect to the choice of p_{missing} and to the optimization of λ

In order to show the robustness of our results with respect to the choice of p_{missing}, we repeated the simulations replacing $p_{\text{missing}} = 0.3$ with $p_{\text{missing}} = 0.2$. Supplementary Fig. 2 compares the original incidence matrix $\mathbf{M}$ with the MC surrogate incidence matrix $\hat{\mathbf{M}}^{(MC)}$, showing that the quality of the reconstruction provided by $\hat{\mathbf{M}}^{(MC)}$ was still good. In particular, in this case the Spearman correlation between $\mathbf{M}$ and $\hat{\mathbf{M}}^{(MC)}$ turned out to be equal to $\rho = 0.46$ (compared to $\rho = 0.53$ obtained for $\lambda = 0.3$).

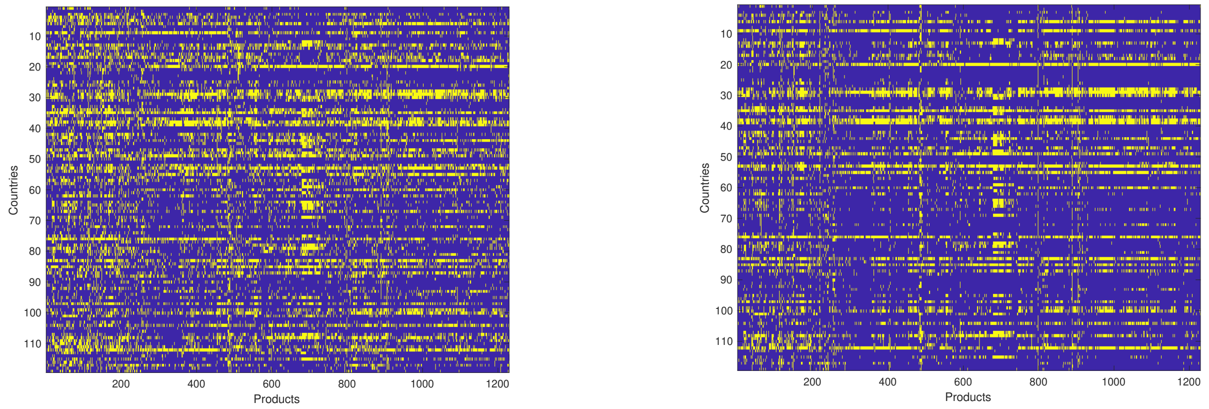

(a) Original incidence matrix $\mathbf{M}$ at the HS-4 level.

(b) MC surrogate incidence matrix $\hat{\mathbf{M}}^{(MC)}$ at the HS-4 level.

Supplementary Figure 2. Similarity between the original incidence matrix $\mathbf{M}$ and the MC surrogate incidence matrix $\hat{\mathbf{M}}^{(MC)}$ when $p_{\text{missing}} = 0.2$ for the year 2018 at the HS-4 level, confirming the robustness of MC prediction performance to variations of p_{missing}.

Moreover, the optimal values of λ selected in the two cases were similar. As an example, Supplementary Fig. 3 provides a comparison between the optimal value of λ selected for one of the repetitions in the two cases of $p_{\text{missing}} = 0.2$ and of

$p_{\text{missing}} = 0.3$.

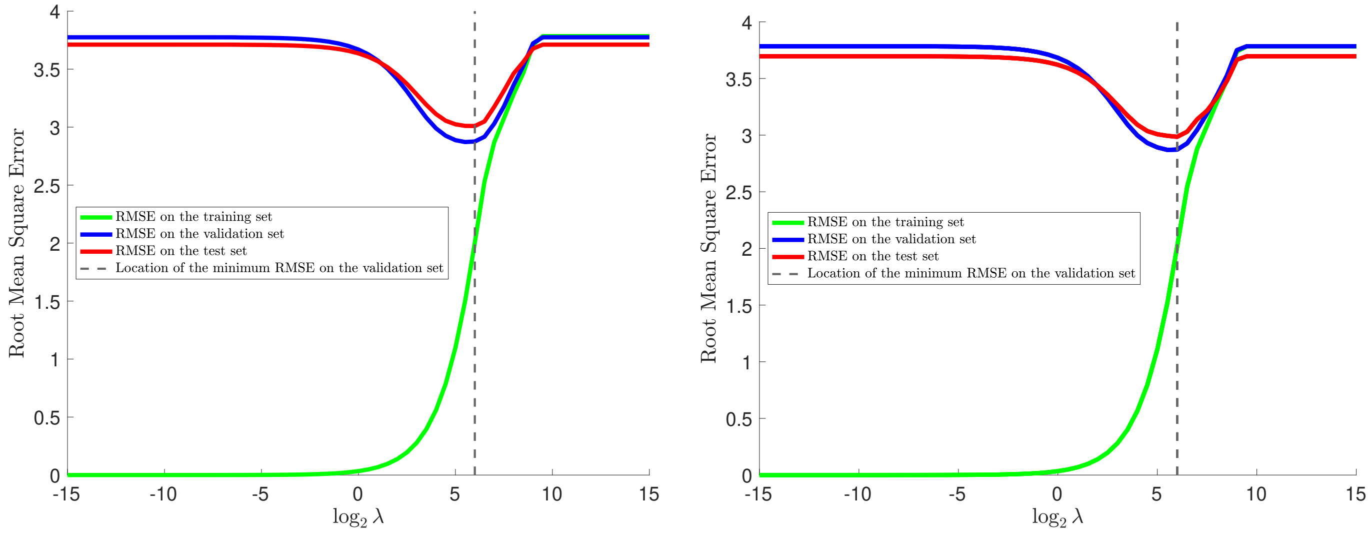

(a) RMSE as a function of $\log_2(\lambda)$ in the case of $p_{\text{missing}} = 0.2$. **(b)** RMSE as a function of $\log_2(\lambda)$ in the case of $p_{\text{missing}} = 0.3$.

Supplementary Figure 3. Similarity in the RMSE optimization for one repetition and the two cases of $p_{\text{missing}} = 0.2$ and $p_{\text{missing}} = 0.3$ at the HS-4 level (year: 2018).

Results similar to those shown in Supplementary Figs. 2 and 3 were obtained for $p_{\text{missing}} = 0.4$.
Finally, we repeated the procedure of construction of the surrogate matrix $\hat{\mathbf{M}}^{(MC)}$ at the HS-4 level without optimizing λ, i.e., fixing λ to $2^{17/2}$, which is the value reported in the right part of Supplementary Fig. 1. Supplementary Fig. 4 shows a strong decrease in the quality of reconstruction of the original incidence matrix $\mathbf{M}$, as a consequence of the suboptimality of this choice of λ. In particular, the Spearman correlation between $\mathbf{M}$ and $\hat{\mathbf{M}}^{(MC)}$ reduced to $\rho = 0.35$.

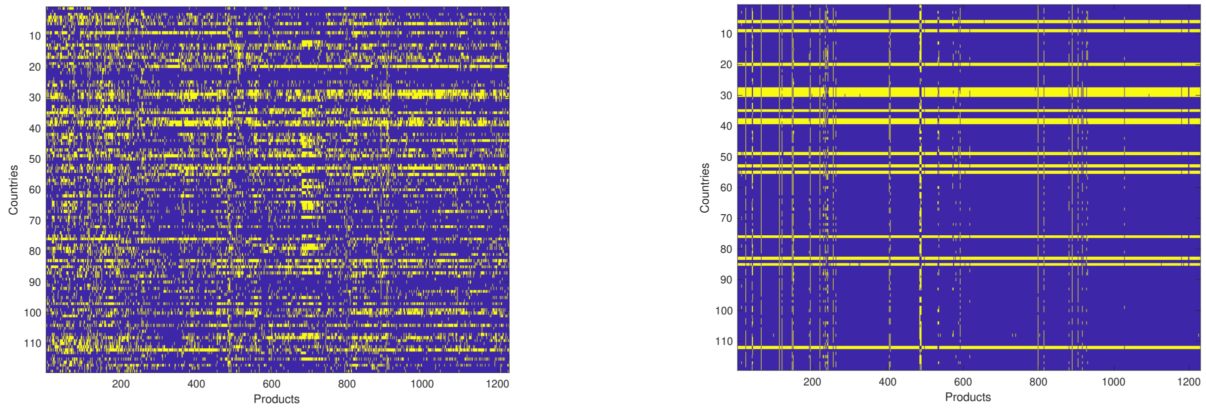

(a) Original incidence matrix $\mathbf{M}$ at the HS-4 level. **(b)** MC surrogate incidence matrix $\hat{\mathbf{M}}^{(MC)}$ at the HS-4 level.

Supplementary Figure 4. Similarity between the original incidence matrix $\mathbf{M}$ and the suboptimal MC surrogate incidence matrix $\hat{\mathbf{M}}^{(MC)}$ for the year 2018 at the HS-4 level (i.e., obtained without optimizing λ), confirming the optimality of the choice of λ made in Fig. 2b in the main text.

Results of the analysis for countries for the year 2018, with products aggregated at the HS-2 level

Supplementary Fig. 5 reports, for the product aggregation level HS-2, results similar to those obtained in the main text for the HS-4 level. For the sake of completeness, we report also results for the false negative rate. Supplementary Fig. 6 provides similar results, restricting to the countries for which both the false negative rate and the false positive are lower than 0.5.

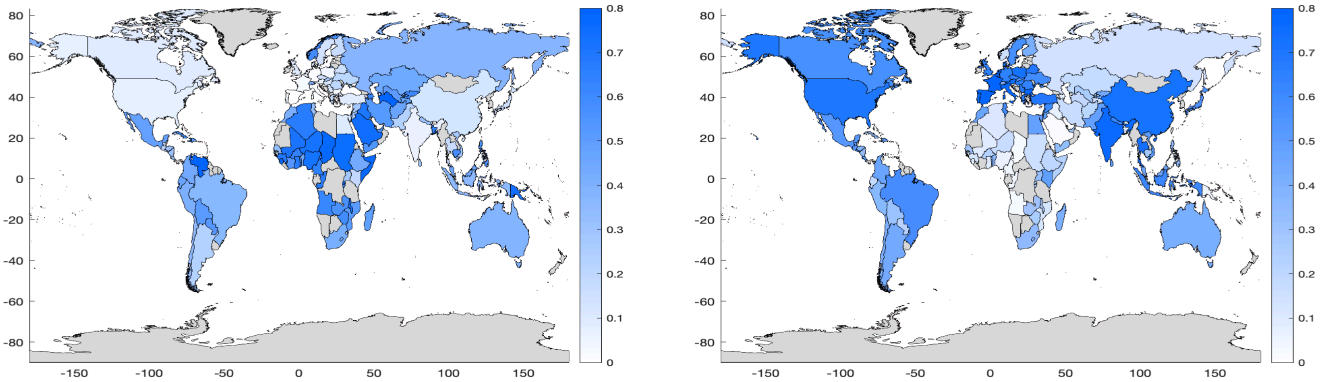

(a) False negative rate fnr_c, reported proportionally to the shade of blue. Countries colored in grey are not considered in the analysis.

(b) False positive rate fpr_c, reported proportionallly to the shade of blue. Countries colored in grey are not considered in the analysis.

Supplementary Figure 5. False negative and false positive rates for countries, obtained by the method reported in Step 7 of our proposed approach for the year 2018 and the HS-2 level of aggregation. The maps were generated using the MATLAB 2012b package `borders`, available for free (upon registration) at the following hyperlink: `https://it.mathworks.com/matlabcentral/fileexchange/50390-borders`.

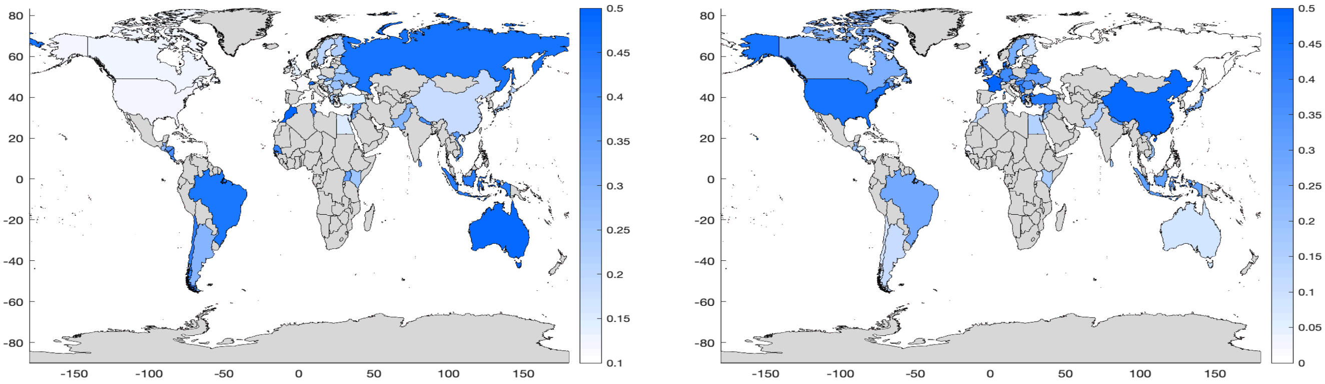

(a) False negative rate fnr_c for countries c having both average false negative and false positive rates lower than 0.5. In the figure, fnr_c is reported proportionally to the shade of blue. Countries colored in grey are not considered in the analysis.

(b) False positive rate fpr_c for countries c having both average false negative and false positive rates lower than 0.5. In the figure, fpr_c is reported proportionally to the shade of blue. Countries colored in grey are not considered in the analysis.

Supplementary Figure 6. False negative and false positive rates for a selection of countries, obtained by the method reported in Step 7 of our proposed approach for the year 2018 and the HS-2 level of aggregation. The maps were generated using the MATLAB 2012b package `borders`, available for free (upon registration) at the following hyperlink: `https://it.mathworks.com/matlabcentral/fileexchange/50390-borders`.

Additionally, Supplementary Tab. 1 reports, for the product aggregation level HS-2, the Kendall rank correlation coefficients τ_k between the ranking produced using GENEPY against the ones produced using either $fnr_{c,hs-2}$ or $fpr_{c,hs-2}$.

	**GENEPY (τ_k)**	**GENEPY (p-value)**
$fnr_{c,hs-2}$	0.1230	0.0575
$fpr_{c,hs-2}$	0.6476	0.0000

Supplementary Table 1. Kendall rank correlation coefficients τ_k and corresponding p-values for the 2018 ranking of countries based on the HS-2 level of aggregation and produced using GENEPY against the 2018 rankings produced respectively by fnr_c, and fpr_c.

Moreover, Supplementary Figs. 7a-7b report the original incidence matrix $\mathbf{M}$ as compared to its MC surrogate $\hat{\mathbf{M}}^{(MC)}$ obtained at the HS-2 level of product aggregation. Also in this case, the two matrices display similar but not identical entries. Thus,

similar conclusions to the ones obtained for the HS-4 case apply.

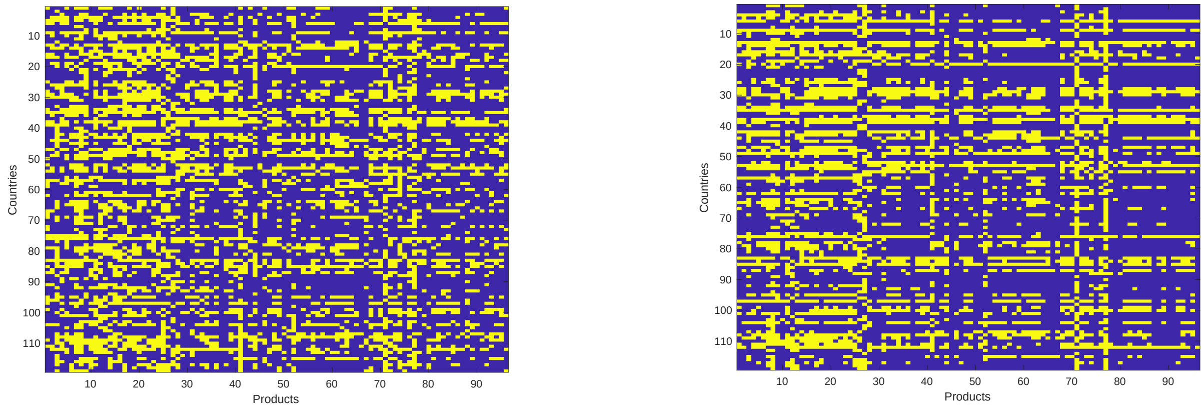

(a) Original incidence matrix $\mathbf{M}$. **(b)** MC surrogate incidence matrix $\hat{\mathbf{M}}^{(MC)}$.

Supplementary Figure 7. Similarity between the original incidence matrix $\mathbf{M}$ and the MC surrogate incidence matrix $\hat{\mathbf{M}}^{(MC)}$ for the year 2018 and the HS-2 level of product aggregation, confirming the good MC prediction performance at a global level.

Results of the analysis for countries for the year 2018, with products aggregated at the HS-6 level

The analysis at the HS-6 level is more computationally expensive, due to the much larger number of products considered. Here we limit to report, respectively in Supplementary Figs. 8 and 9, a comparison between the original incidence matrix M and the MC surrogate incidence matrix $(\hat{\mathbf{M}}^{\top})^{(MC)}$ for the year 2018 at the HS-6 level of product aggregation (obtained Spearman correlation $\rho = 0.52$), and the associated global ROC curve. In this case, following Hidalgo (2021)[4], only countries having less than 1 million inhabitants have been removed from the dataset.

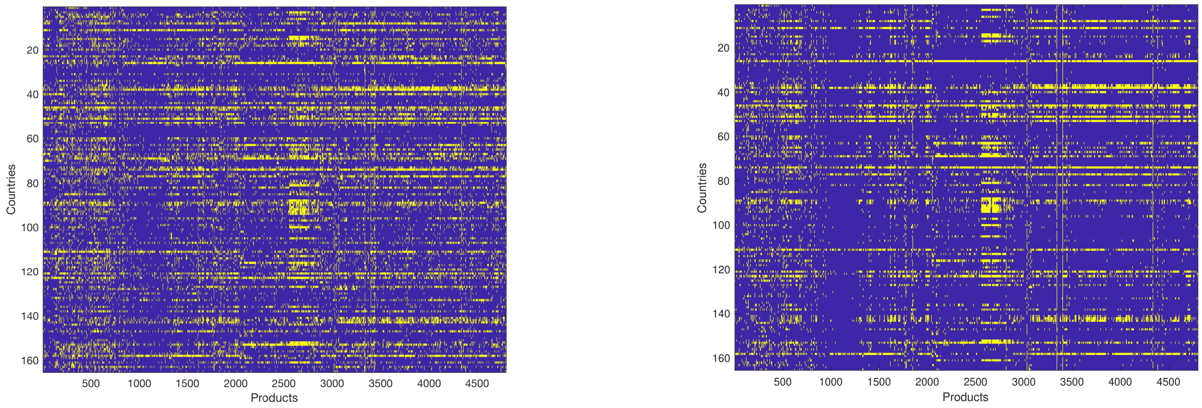

(a) Original incidence matrix $\mathbf{M}$. **(b)** MC surrogate incidence matrix $\hat{\mathbf{M}}^{(MC)}$.

Supplementary Figure 8. Similarity between the original incidence matrix $\mathbf{M}$ and the MC surrogate incidence matrix $\hat{\mathbf{M}}^{(MC)}$ for the year 2018 at the HS-6 level, confirming the good MC prediction performance at a global level.

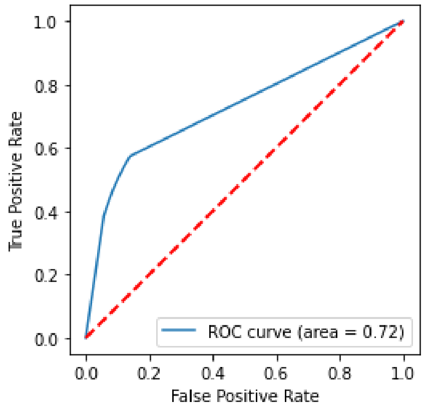

Supplementary Figure 9. Global ROC curve constructed starting from the matrix $\overline{\mathbf{M}}^{(MC)}$, for the year 2018 and the HS-6 level of product aggregation. A reference dotted line passing through the origin with slope 1 is also reported.

As a further step, we also compare the classification performance achieved in link prediction by the binary classifier based on the MC surrogate incidence matrix $\overline{\mathbf{M}}^{(MC)}$ with alternative machine learning techniques used in Tacchella et al. (2021)[12]. The comparison is fair as both analyses refer to the year 2018 and to the HS-6 level of product aggregation. The best F1 score of MC is 0.73, higher than the similar metrics for other machine learning methods reported in Tacchella et al. (2021)[12].

MONEY index for the years 2005 and 2014

In the following, results similar to those obtained in the main text are reported for the years 2005 and 2014. For those years, the global AUC values are 0.76 for year 2005 and 0.80 for year 2014. Since the two categories $RCA \geq 1$ and $0 \leq RCA < 1$ are unbalanced, we also computed the BACC which amounts to 0.74 for 2005 and to 0.75 for 2014 (at the 0.5 threshold). Supplementary Fig. 10 displays the MONEY index for the years 2005 and 2014.

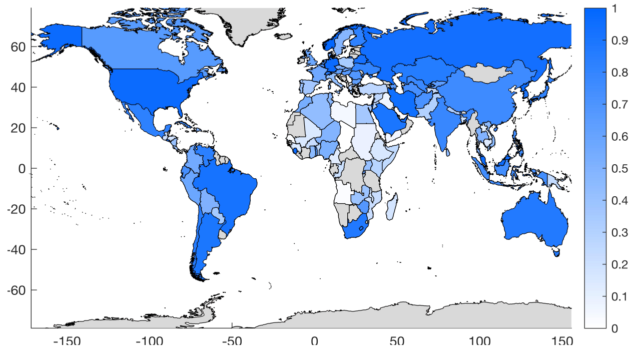

(a) Values of the MONEY index for the year 2005 at the HS-4 level of aggregation.

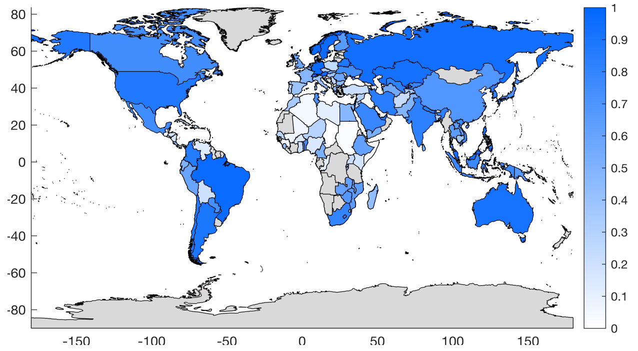

(b) Values of the MONEY index for the year 2014 at the HS-4 level of aggregation.

Supplementary Figure 10. Values of the MONEY index for the years 2005 and 2014 at the HS-4 level of aggregation. The maps were generated using the MATLAB 2012b package `borders`, available for free (upon registration) at the following hyperlink: https://it.mathworks.com/matlabcentral/fileexchange/50390-borders.

Results are similar to those obtained in the main analysis for year 2018. However, some differences emerge. Specifically, Russia and East Asia appear to be more complex in 2005 and 2014 than in 2018.

To further corroborate our findings, we repeat our analysis at the HS-2 level of product aggregation. Supplementary Fig. 11 reports false negative and false positive rates for the two years. Supplementary Tab. 2 reports Kendall rank correlation coefficients between the ranking of GENEPY and the rankings produced respectively by the false negative rate ($fnr_{c,t}$) and the false positive rate ($fpr_{c,t}$).

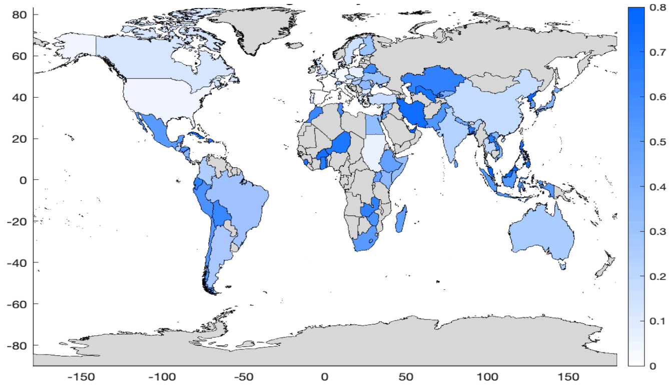

(a) False negative rate fnr_c, reported proportionally to the shade of blue for the year 2005. Countries colored in grey are not considered in the analysis.

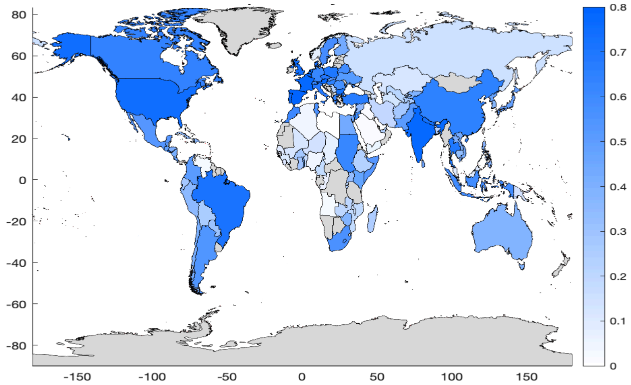

(b) False positive rate fpr_c, reported proportionally to the shade of blue for the year 2005. Countries colored in grey are not considered in the analysis.

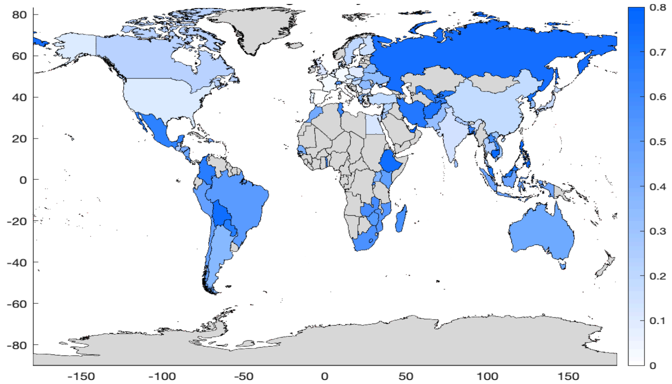

(c) False negative rate fnr_c, reported proportionally to the shade of blue for the year 2014. Countries colored in grey are not considered in the analysis.

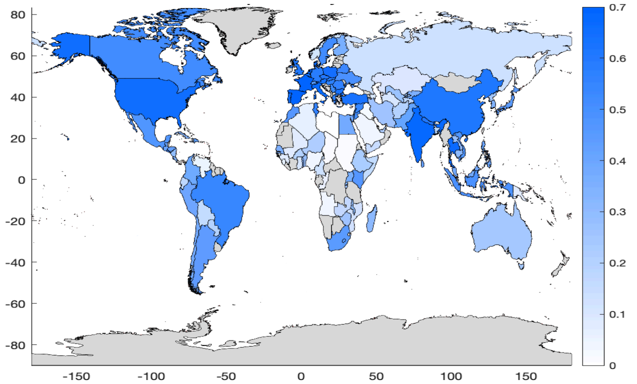

(d) False positive rate fpr_c, reported proportionally to the shade of blue for the year 2014. Countries colored in grey are not considered in the analysis.

Supplementary Figure 11. False negative and false positive rates for countries, obtained by the method reported in Step 7 of our proposed approach for the years 2005 and 2014, with products aggregated at the HS-2 level. The maps were generated using the MATLAB 2012b package `borders`, available for free (upon registration) at the following hyperlink: https://it.mathworks.com/matlabcentral/fileexchange/50390-borders.

	**GENEPY**$_t$ **(**τ_k**)**	**GENEPY**$_t$ **(**p**-value)**
$fnr_{c,2005}$	0.2018	0.0020
$fpr_{c,2005}$	0.6212	0.0000
$fnr_{c,2014}$	0.1936	0.0031
$fpr_{c,2014}$	0.6165	0.0000

Supplementary Table 2. τ_k and relative p-values for the rankings produced using GENEPY against the ranking produced respectively by $fnr_{c,t}$ and $fpr_{c,t}$, for the years $t = 2005$ and $t = 2014$, with products aggregated at the HS-2 level.

Application of the analysis to the products at the HS-2 level

The same analysis made in the main text for the countries has been repeated for the products, still referring to the year 2018. This is obtained simply by replacing at the beginning of the analysis the **RCA** matrix with its transpose. Notice that this analysis, as some of the analyses reported in this Supplementary material, was made at the HS-2 level for computational time reasons. The results obtained at the HS-2 level, however, correlated at the 95% with the ones obtained at the HS-4 level. Supplementary Figs. 12a-12d display respectively, on the main diagonal of each matrix reported, and for a subset of product codes,

- the false negative rate fnr_p for each product p (Supplementary Fig. 12a);

- the false positive rate fpr_p for each product p (Supplementary Fig. 12b);
- the false negative rate fnr_p, for the subset of products p for which both fnr_p and fpr_p are lower than 0.5 (Supplementary Fig. 12c);
- the false positive rate fpr_p, for the subset of products p for which both fnr_p abd fpr_p are lower than 0.5 (Supplementary Fig. 12d).

In all these cases, the products have been ordered increasingly with respect to the (either false positive or false negative) rate.

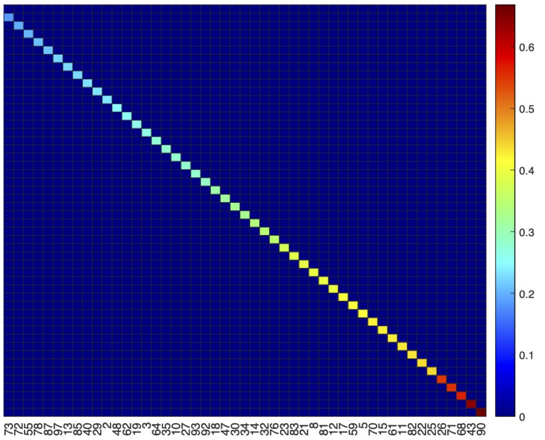

(a) False negative rate fnr_p for the products, reported as a function of the color shade from blue (associated with the lowest value) to red (associated with the highest value).

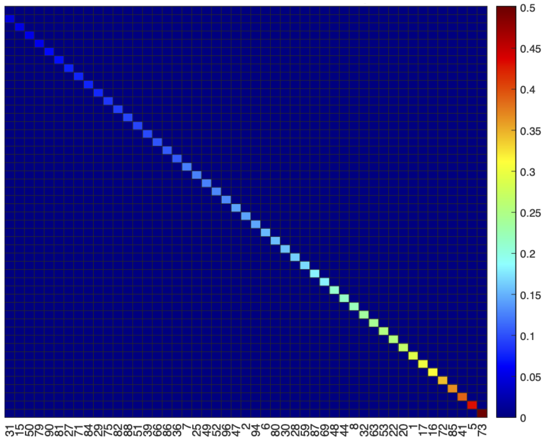

(b) False positive rate fpr_p for the products, reported as a function of the color shade from blue (associated with the lowest value) to red (associated with the highest value).

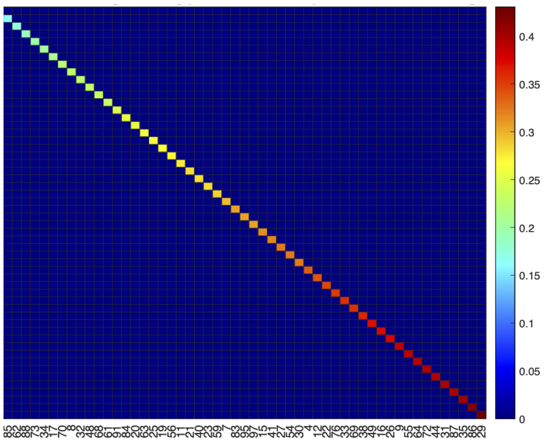

(c) False negative rate fnr_p for the products having both $fnr_c < 0.5$ and $fpr_c < 0.5$. Values of fnr_c are reported as a function of the color shade from blue (associated with the lowest value) to red (associated with the highest value).

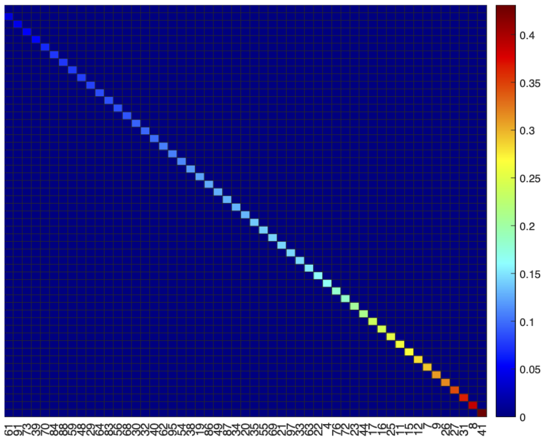

(d) False positive rate fpr_p for the products having both $fnr_c < 0.5$ and $fpr_c < 0.5$. Values of fpr_c are reported as a function of the color shade from blue (associated with the lowest value) to red (associated with the highest value).

Supplementary Figure 12. False negative and false positive rates for products, obtained by the method reported in Step 7 of our proposed approach for the year 2018 and the HS-2 level.

The correspondence between product codes and their names is reported in Supplementary Tab. 3. The names are reported only for the HS-2 level for a better readability.

Row index	Product name
1	Live animals
2	Meat and edible meat offal
3	Fish and crustaceans, molluscs and other aquatic invertebrates
4	Dairy produce
5	Products of animal origin, not elsewhere specified or included
6	Live trees and other plants
7	Edible vegetables and certain roots and tubers
8	Edible fruit and nuts;
9	Coffee, tea, mate and spices
10	Cereals
11	Products of the milling industry;
12	Oil seeds and oleaginous fruits;
13	Lac; gums, resins and other vegetable saps and extracts
14	Vegetable plaiting materials
15	Animal or vegetable fats and oils and their cleavage products
16	Preparations of meat, of fish or of crustaceans
17	Sugars and sugar confectionery
18	Cocoa and cocoa preparations
19	Preparations of cereals, flour, starch or milk; pastrycooks' products
20	Preparations of vegetables, fruit, nuts or other parts of plants
21	Miscellaneous edible preparations
22	Beverages, spirits and vinegar
23	Residues and waste from the food industries; prepared animal fodder
24	Tobacco and manufactured tobacco substitutes
25	Salt; sulphur
26	Ores, slag and ash
27	Mineral fuels, mineral oils and products of their distillation; bituminous substances
28	Inorganic chemicals; organic or inorganic compounds of precious metals, of rare-earth metals, of radioactive elements or of isotopes
29	Organic chemicals
30	Pharmaceutical products
31	Fertilisers
32	Tanning or dyeing extracts
33	Essential oils and resinoids
34	Soap, organic surface-active agents, washing preparations, lubricating preparations, artificial waxes...
35	Albuminoidal substances
36	Explosives, fireworks
37	Photographic or cinematographic goods
38	Miscellaneous chemical products
39	Plastics and articles thereof
40	Rubber and articles thereof
41	Raw hides and skins
42	Articles of leather; saddlery and harness
43	Furskins and artificial fur
44	Wood and articles of wood
45	Cork and articles of cork
46	Manufactures of straw, of esparto or of other plaiting materials
47	Pulp of wood or of other fibrous cellulosic material
48	Paper and paperboard
49	Printed books, newspapers, pictures and other products of the printing industry
50	Silk
51	Wool, fine or coarse animal hair
52	Cotton
53	Other vegetable textile fibres
54	Sewing thread of man-made filaments
55	Man-made staple fibres
56	Wadding, felt and nonwovens
57	Carpets and other textile floor coverings
58	Special woven fabrics
59	Impregnated, coated, covered or laminated textile fabrics
60	Knitted or crocheted fabrics
61	Articles of apparel and clothing accessories, knitted or crocheted
62	Articles of apparel and clothing accessories, not knitted or crocheted
63	Other made up textile articles
64	Footwear, gaiters and the like; parts of such articles
65	Headgear and parts thereof
66	Umbrellas, sun umbrellas and similar articles
67	Prepared feathers and down and articles made of feathers or of down
68	Articles of stone, plaster, cement, asbestos, mica or similar materials
69	Ceramic products
70	Glass and glassware
71	Natural or cultured pearls, precious or semi-precious stones, precious metals
72	Iron and steel
73	Articles of iron or steel
74	Copper and articles thereof
75	Nickel and articles thereof
76	Aluminium and articles thereof
78	Lead and articles thereof
79	Zinc and articles thereof
80	Tin and articles thereof
81	Other base metals; cermets; articles thereof
82	Tools, implements, cutlery, spoons and forks, of base metal; parts thereof of base metal
83	Miscellaneous articles of base metal
84	Nuclear reactors, boilers, machinery and mechanical appliances
85	Electrical machinery and equipment and parts thereof
86	Railway or tramway locomotives, rolling-stock and parts thereof
87	Vehicles other than railway or tramway rolling-stock, and parts and accessories thereof
88	Aircraft, spacecraft, and parts thereof
89	Ships, boats and floating structures
90	Optical, photographic, cinematographic, measuring, checking, precision, medical or surgical instruments and apparatus; parts and accessories thereof
91	Clocks and watches and parts thereof
92	Musical instruments; parts and accessories of such articles
93	Arms and ammunition; parts and accessories thereof
94	Furniture; bedding, mattresses, mattress supports, cushions and similar stuffed furnishings
95	Toys, games and sports requisites; parts and accessories thereof
96	Miscellaneous manufactured articles
97	Works of art, collectors' pieces, and antiques

Supplementary Table 3. Product codes and corresponding names at the HS-2 level.

Finally, we provide in Supplementary Tab. 4 the ranking of products by false positive rate for the year 2018. We report here only the top 10 product categories (for both the analyses at the HS-2 and HS-4 levels).

fpr, **HS-2**	fpr, **HS-4**
Lead and articles thereof	Pharmaceutical products
Pearls, precious stones	Sugars and sugar confectionery
Fertilisers	Iron and steel
Copper and articles thereof	Lead and articles thereof
Zinc and articles thereof	Mineral fuels, mineral oils and products of their distillation; bituminous substances; mineral waxes
Tobacco and manufactured tobacco substitutes	Natural or cultured pearls, precious or semi-precious stones, precious metals
Mineral fuels, mineral oils (and related)	Oil seeds and oleaginous fruits; miscellaneous grains, seeds and fruit; industrial or medicinal plants
Other base metals; cermets; articles thereof	Vehicles other than railway or tramway rolling-stock, and parts and accessories thereof
Silk	Aluminium and articles thereof

Supplementary Table 4. Top 10 products by false positive rate; HS-2 and HS-4 levels of aggregation; year: 2018.

At both levels of aggregation, the analysis was conducted in this case by comparing the matrix of true values with a matrix of predicted ones, obtained through 500 simulations. This was done in order to provide a robustness check for the results. Specifically, we created 500 predicted matrices by applying the MC algorithm several times (after transposing the original matrix). Out of those 500 matrices, we recovered a single predicted matrix by taking the median for each element of the 500 predicted matrices (when it was in the test set), then discretizing it using 1 as threshold for the RCA value. Finally, the latter

discretized matrix was used to compute the false-positive rates by products.
As shown in Supplementary Tab. 4, the false positives do not concentrate on the same products at the two aggregation levels, though some similarities emerge.

Application of a variation of the analysis to countries, based on the entry-wise logarithm of the original RCA matrix for the year 2018, with products aggregated at the HS-4 level

In the following, a variation of our analysis is applied to countries. In this variation, instead of discretizing the elements of the original **RCA** matrix, they are replaced by their natural logarithm (no issues arise when taking the natural logarithm, because no 0 or negative entries are present in that matrix; moreover, entries originally equal to *NaN* are never included in the training, validation or test sets). Then, the rest of the proposed method is unchanged with respect to the main text, where the discretization method has been preferred, because it generates values more symmetrically distributed around 0.
The first motivation for the natural logarithm analysis is that the original data are continuous. Yet, in the main analysis, we categorized the original data. A further reason is that matrix completion works typically better when the elements of the matrix have similar order of magnitude. However, RCA values are well-known to be heavy tailed. Thus, as a robustness check of the previous analysis, we reduced the scale by taking the natural logarithm.
Supplementary Fig. 13 reports, for this variation of analysis and for the product aggregation level HS-4, results similar to those obtained in Supplementary Figs. 5a and 5b for the original analysis and for the product aggregation level HS-2.

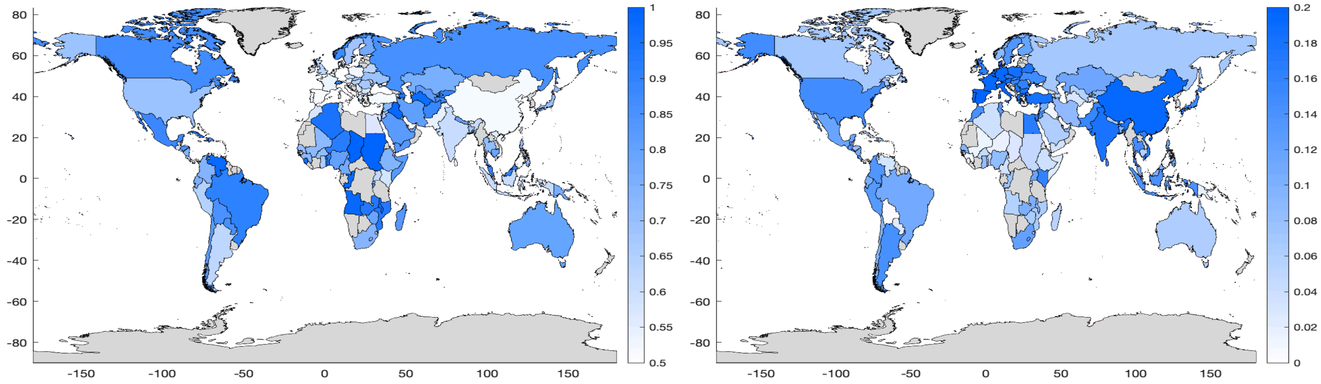

(a) False negative rate fnr_c, reported proportionally to the shade of blue for the year 2018 and the product aggregation level HS-4, for the case in which the entry-wise natural logarithm of the original **RCA** matrix is employed by the proposed method. Countries colored in grey are not considered in the analysis.

(b) False positive rate fpr_c, reported proportionally to the shade of blue for the year 2018 and the product aggregation level HS-4, for the case in which the entry-wise natural logarithm of the original **RCA** matrix is employed by the proposed method. Countries colored in grey are not considered in the analysis.

Supplementary Figure 13. False negative and false positive rates for countries for the year 2018 and the product aggregation level HS-4, for the case in which the entry-wise natural logarithm of the original **RCA** matrix is employed by the proposed method. The maps were generated using the MATLAB 2012b package `borders`, available for free (upon registration) at the following hyperlink:
https://it.mathworks.com/matlabcentral/fileexchange/50390-borders.

Similarly, Supplementary Tab. 5 finds, for this variation of analysis and for the product aggregation level HS-4, results similar to those obtained in Supplementary Tab. 2 for the original analysis and for the product aggregation level HS-2.

	**GENEPY (τ_k)**	**GENEPY (p-value)**
$fnr_{c,log,hs-4}$	-0.2569	0.0000
$fpr_{c,log,hs-4}$	0.5903	0.0000

Supplementary Table 5. τ_k and relative p-values for the rankings produced using GENEPY against the ranking produced respectively by $fnr_{c,t}$ and $fpr_{c,t}$, for the product aggregation level HS-4, for the case in which the entry-wise natural logarithm of the original **RCA** matrix is employed by the original analysis.

Comparison of MONEY with other country complexity indices for the years 2005 ad 2014

Supplementary Figs. 14 and 15 report comparison tables among the rankings provided by the various country complexity indices at the HS-4 level, taking MONEY as the reference point. For space reasons, the two figures refer to a selection of countries and, respectively, to the years 2005 and 2014.

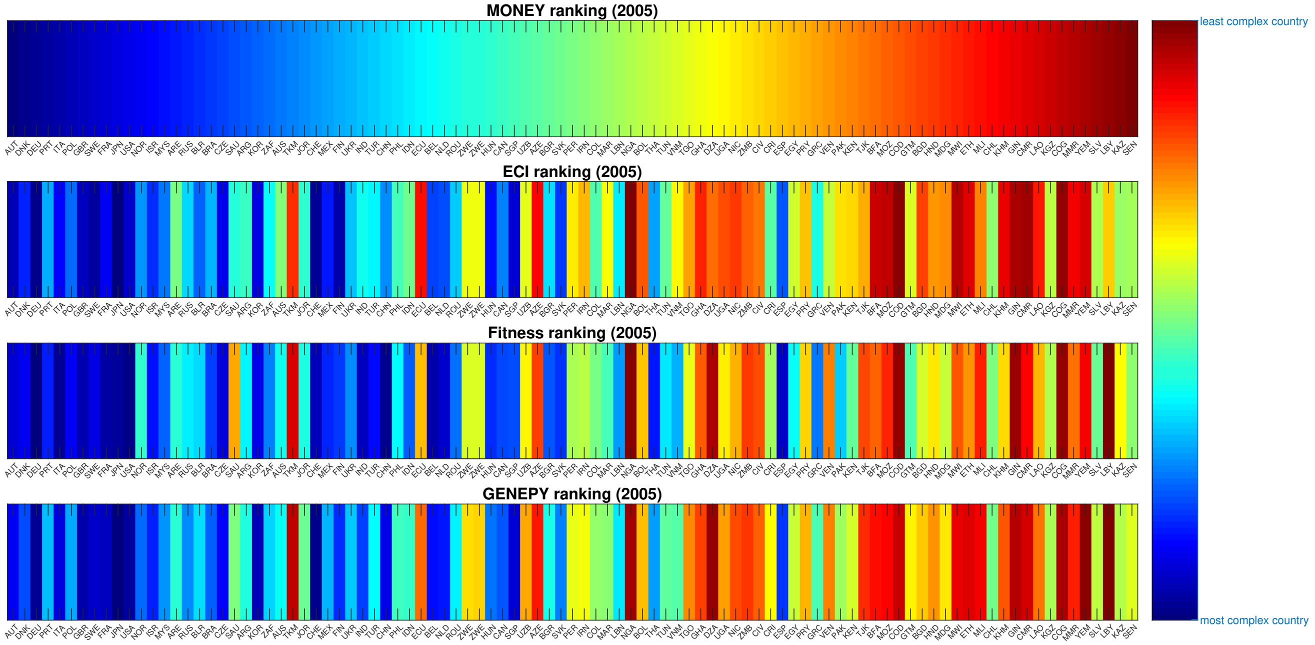

Supplementary Figure 14. Comparison table for various country complexity indices in 2005 (HS-4 level).

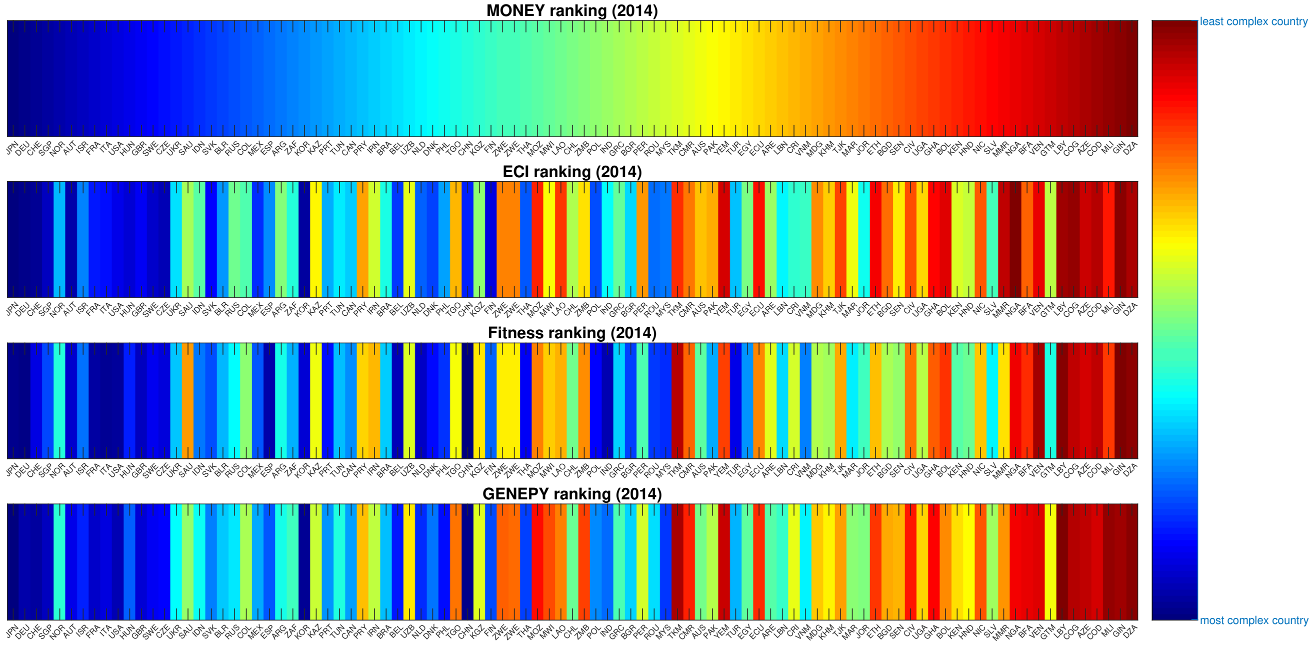

Supplementary Figure 15. Comparison table for various country complexity indices in 2014 (HS-4 level).

Finally, Supplementary Tab. 6 reports the values assumed by the Kendall rank correlation coefficient τ_k when comparing the rankings (at the HS-4 level) provided by ECI, Fitness, GENEPY and MONEY, for the years 2005 and 2014.

		Economic complexity and GDP per capita, PPP				
**Year**	**Index**	ECI	Fitness	GENEPY	MONEY	GDP per capita, PPP
2005	ECI	1	0.75	0.83	0.53	0.60
	Fitness	.	1	0.85	0.50	0.49
	GENEPY	.	.	1	0.54	0.57
	MONEY	.	.	.	1	0.52
2014	ECI	1	0.76	0.82	0.58	0.54
	Fitness	.	1	0.83	0.50	0.46
	GENEPY	.	.	1	0.57	0.56
	MONEY	.	.	.	1	0.48

Supplementary Table 6. Kendall rank correlation coefficient τ_k for economic complexity indices (ECI, Fitness, GENEPY and MONEY) and the GDP per capita, PPP. World trade at the HS-4 level. Years: 2005 and 2014.

For these two years, the MONEY index has a slightly lower Kendall rank correlation coefficient τ_k with the ranking provided by GDP per capita, PPP than ECI and GENEPY (but still higher than Fitness). It is important to state, however, that complexity indices have been introduced in the literature to overcome the classical growth theory which was heavily based on GDP per capita and on its variations, in order to take into account other (hidden) factors contributing to the overall complexity of a country (Hidalgo, 2021[4]). For this reason, we find the slightly lower Kendall rank correlation coefficient τ_k achieved by MONEY as indicative of the possible discovery of hidden production factors, not taken into account both by previous complexity indices and by the GDP per capita PPP.

References

1. Foucart, S., Needell, D., Plan Y., & Wootters, M. (2017). De-biasing low-rank projection for matrix completion. Proceedings of SPIE, 10394, Wavelets and Sparsity XVII, 1039417, 13 pages.

2. Hastie, T., Tibshirani, R., & Friedman, J. (2009). The elements of statistical learning: data mining, inference, and prediction. Springer.

3. Hastie, T., Tibshirani, R., & Wainwright, M. (2015). Statistical learning with sparsity: the Lasso and generalizations.

4. Hidalgo, C. A. (2021). Economic complexity theory and applications. Nature Reviews Physics, 3, pp. 92–113.

5. Hidalgo, C. A., & Hausmann, R (2009). The building blocks of economic complexity. Proceedings of the National Academy of Sciences, 106, pp. 10570–10575.

6. Li, C., & Zhou, H. (2017). Svt: Singular value thresholding in MATLAB. Journal of Statistical Software, 81(2), DOI: 10.18637/jss.v081.c02.

7. Ma, W., & Chen, G. H. (2019). Missing not at random in matrix completion: the effectiveness of estimating missingness probabilities under a low nuclear norm assumption. In Advances in Neural Information Processing Systems 32, Proceedings of the 33rd Conference on Neural Information Processing Systems (NeurIPS 2019), Vancouver, Canada.

8. Mazumder, R., Hastie, T., & Tibshirani, R. (2010). Spectral regularization algorithms for learning large incomplete matrices. Journal of Machine Learning Research, 11, pp. 2287–2322.

9. Sciarra, C., Chiarotti, G., Laio, F., & Ridolfi, L. (2018). A change of perspective in network centrality. Scientific Reports, 8, article no. 15269.

10. Sciarra, C., Chiarotti, G., Ridolfi, G., & Laio, F. (2020). Reconciling contrasting views on economic complexity. Nature Communications, 11, article no. 3352.

11. Tacchella, A., Cristelli, M., Caldarelli, G., Gabrielli, A., & Pietronero, L. (2012). A new metrics for countries' fitness and products' complexity. Scientific Reports, 2, article no. 723.

12. Tacchella, A., Zaccaria, A., Miccheli, M., & Pietronero, L. (2021). Relatedness in the era of machine learning, ArXiv preprint arXiv:2103.06017.

13. Tibshirani, R. (1996). Regression shrinkage and selection via the Lasso. Journal of the Royal Statistical Society. Series B (Methodological), 58(1), pp. 267–288.
